# Predictive value of PfEMP1 antibody profiles for the course of controlled human malaria infections

**DOI:** 10.1371/journal.ppat.1014377

**Published:** 2026-06-24

**Authors:** Yannick D. Höppner, Ralf Krumkamp, Hikaru Nagaoka, Charlotte Wapler, Hannah Honner, Louise Turner, Jean Claude Dejon-Agobé, Yabo J. Honkpehedji, Jeannot Fréjus Zinsou, Jana Held, Meral Esen, Heidrun von Thien, Iris Bruchhaus, B. Kim Lee Sim, Stephen L. Hoffman, Peter G. Kremsner, Tim-Wolf Gilberger, Bertrand Lell, Takafumi Tsuboi, Thomas Lavstsen, Eizo Takashima, Benjamin Mordmüller, Anna Bachmann

**Affiliations:** 1 Bernhard Nocht Institute for Tropical Medicine, Hamburg, Germany; 2 German Center for Infection Research (DZIF), Hamburg-Borstel-Lübeck-Riems, Germany; 3 Proteo-Science Center, Ehime University, Matsuyama, Japan; 4 Center for Medical Parasitology, Department of Immunology and Microbiology, University of Copenhagen, Copenhagen, Denmark; 5 Department of Infectious Diseases, Copenhagen University Hospital, Copenhagen, Denmark; 6 Centre de Recherches Médicales de Lambaréné, Lambaréné, Gabon; 7 Leiden University Center for Infectious Diseases (LUCID), Leiden University Medical Center (LUMC), Leiden, the Netherlands; 8 Institute of Tropical Medicine, University of Tübingen, Tübingen, Germany; 9 German Center for Infection Research (DZIF), Tübingen, Germany; 10 Centre for Structural Systems Biology, Hamburg, Germany; 11 Biology Department, University of Hamburg, Hamburg, Germany; 12 Sanaria Inc., Medical Center Drive, Rockville, Maryland, United States of America; 13 Department of Medicine I, Division of Infectious Diseases and Tropical Medicine, Medical University of Vienna, Vienna, Austria; 14 Radboud University Medical Center, Radboud Institute of Molecular Life Sciences, Department of Medical Microbiology, The Netherlands; Universidade de São Paulo: Universidade de Sao Paulo, BRAZIL

## Abstract

Antibody-mediated immunity directed against *Plasmodium falciparum* erythrocyte membrane protein 1 (PfEMP1) is an important immune mechanism for protection against clinical malaria and control of parasitemia. Current studies on PfEMP1-based immunity are constrained by the protein’s high molecular weight, complex multidomain architecture, and extensive polymorphism. Controlled human malaria infection (CHMI) studies uniquely enable the assessment of strain-specific PfEMP1 immunity prior to exposure and its correlation with infection outcomes using the same parasite strain. In this study, different serological assays targeting both full-length native PfEMP1 and individual domains were applied to plasma samples obtained prior to challenge infection at study baseline from 25 individuals enrolled in a CHMI study of PfSPZ Challenge (NF54), that included malaria-naïve Europeans and lifelong malaria-exposed Africans with varying degrees of immunity. All assays showed strong predictive value of both PfEMP1 antibody levels and breadth at study baseline for CHMI outcome. A random forest machine learning analysis of antibody recognition profiles, measured by AlphaScreen nearly covering all extracellular PfEMP1 domains of the NF54-CHMI parasite strain, suggested that a broad antibody repertoire, including antibodies against the most diversified group B PfEMP1s, represents a discriminative feature distinguishing volunteers with high versus low susceptibility to CHMI. In parasites re-isolated from the volunteers, dominant *var* transcripts encoded PfEMP1 variants are not recognized by the individuals’ pre-existing antibodies at study baseline. Given that the CHMI strain NF54 predominantly expresses B-type *var* genes at the onset of blood-stage infection, effective control of CHMI was associated with a broad antibody repertoire, particularly including antibodies targeting B-type PfEMP1. In line with the “hole-in-the-wall” theory, this likely reflects an antibody-mediated restriction of the parasite population emerging from the liver to parasites expressing PfEMP1 variants not previously encountered by the host immune system.

## Author summary

Malaria remains a major global health burden, and effective control depends on a detailed understanding of host immune responses to *Plasmodium falciparum*. In this study, we investigated antibody responses against *P. falciparum* erythrocyte membrane protein 1 (PfEMP1), the key virulence factor that mediates cytoadherence and antigenic variation during the blood-stage of infection. PfEMP1 is characterized by extensive antigenic diversity and structural complexity, which have impeded strain-specific immunological analyses so far. Using plasma samples from volunteers undergoing controlled human malaria infection (CHMI) by injection of PfSPZ Challenge with a reference parasite strain, we analyzed the relationship between naturally pre-acquired PfEMP1 antibody responses and infection control. Individuals with a higher level and broader repertoire of PfEMP1 antibodies demonstrated improved control of infection. Moreover, integrated analyses of parasite *var* gene expression and host antibody profiles revealed patterns consistent with immune selection. Parasites preferentially expressed PfEMP1 variants that the host had not previously encountered, effectively exploiting gaps in the existing antibody repertoire to establish the infection. Our findings suggest that a broad antibody response contribute to limiting blood-stage infection and highlight the dynamic interplay between parasite antigenic variation and host immunity.

## Introduction

Malaria remains one of the most significant global health challenges, particularly in children under five years of age and pregnant women [1]. In humans, malaria is caused by several species of *Plasmodium* (*P.*), with *P. falciparum* causing by far the highest burden of disease. In high transmission areas, immunity to malaria is acquired gradually, with children first developing “antidisease immunity” after a few infections, which reduces morbidity despite high parasite levels, followed by more slowly acquired “antiparasite immunity” that limits high parasitemia and associated illness [2]. Because sterilizing immunity is probably never fully achieved, adults often remain asymptomatic carriers (reviewed in [2,3]). Generally, naturally acquired immunity (NAI) to malaria remains poorly understood, but antibody responses against blood-stage antigens were identified by passive transfer experiments as essential for control of parasitemia and prevention of clinical symptoms [4,5].

The main targets of the blood stage antibody response are merozoite surface and secreted antigens as well as the variant surface antigen *P. falciparum* erythrocyte membrane protein 1 (PfEMP1), the key virulence factor mediating immune evasion [6–9]. Parasites adhere via PfEMP1 to the host cell receptors presented on the microvasculature and leave the blood circulation, thereby preventing splenic passage and removal by immune cells. Parasite sequestration, along with excessive inflammation and vascular endothelial dysfunction, is considered central to the pathogenesis of severe malaria [10,11]. Each parasite genome is equipped with about 60 *var* genes encoding different PfEMP1 variants. While *var* gene expression has long been considered mutually exclusive, with a single dominant PfEMP1 variant expressed on the surface of the infected red blood cell (iRBC), recent work suggests that *var* expression may be more flexible than previously thought, and that co-expression of multiple *var* genes can occur at the single-cell level [12]. During asexual replication, switching to expression of different *var* genes in parasite progeny facilitates antigenic variation, giving rise to the characteristic fluctuations in parasitemia throughout the infection [13].

PfEMP1 are large, polymorphic proteins composed of a variable number (between 2 and 10) of alternating Duffy binding-like (DBL; subclasses: α–ζ, pam) and cysteine-rich interdomain region (CIDR; subclasses: α–δ, pam) domains, a transmembrane region and a conserved, intracellular located acid terminal segment (ATS). Its multidomain structure enables a single PfEMP1 to bind to multiple host receptors [14,15]. Despite their high polymorphism, PfEMP1 share a semi-conserved N-terminal head-structure consisting of an N-terminal segment (NTS), a DBLα domain, and a CIDR domain. Within this, the CIDR domain confers a mutually exclusive binding signature of the PfEMP1 to either CD36, EPCR, CSA, or a yet “unknown A” receptor [16,17]. These N-terminal protein receptor-binding signatures also align closely with the structuring of the *var* gene family, with groups A, B, C, and E differing in their 5′ UTR regions, chromosomal positions, and transcriptional orientations [18–21]. Group A *var* genes are subtelomerically located and encode the largest proteins (300–400 kDa) that typically contain a DBLα1 domain in tandem with a CIDR domain that bind either EPCR (CIDRα1.4–7) or “unknown A” receptors (CIDRβ/γ/δ) followed by multiple DBL and CIDR domains of other subclasses. Expression of A-type PfEMP1 and both PfEMP1 binding signatures, EPCR and “unknown A”, have been associated with severe malaria [22–28]. In contrast, *var* groups B and C encode smaller PfEMP1 variants (170–270 kDa), typically with four extracellular domains starting with a DBLα0–CIDRα2–6 head structure that mediates binding to CD36, a receptor broadly expressed on vascular endothelium, followed by a paired DBLδ and CIDRβ/γ/δ domain. Group B *var* genes are located in the most subtelomeric regions of the chromosomes, whereas group C genes are found in central chromosomal clusters. Additionally, variants that share features of two groups form the chimeric B/A (also known as DC8-PfEMP1 binding EPCR [29]) and B/C subgroups. The more conserved variant VAR2CSA constitutes group E, specifically binds CSA in the placenta via its CIDRpam domain, and plays a key role in pregnancy-associated malaria [30]. Other inter-strain conserved PfEMP1 variants belong to group A, including the pseudogene VAR1 and the short VAR3, but their specific functions remain unknown.

PfEMP1-specific IgG antibodies exhibit both neutralizing and opsonizing activity [31], and can interfere with sequestration of infected erythrocytes (iEs) by inhibiting endothelial binding, thereby facilitating splenic clearance of parasitized cells. Opsonization of iEs may result in antibody-dependent cellular phagocytosis (ADCP) or antibody-dependent cellular cytotoxicity (ADCC) [32]. An interesting feature of malaria immunity is that immunity to severe malaria develops at a much faster rate than that to uncomplicated malaria [2]. Therefore, naturally acquired antibodies against a restricted and more conserved group of PfEMP1 variants have been proposed to confer protection against severe disease [33,34]. These are most likely A-type PfEMP1 proteins, particularly those binding EPCR or the “Unknown A” receptor. This is supported by three observations: (i) recombinant proteins derived from group A and B/A PfEMP1 are more frequently recognized than domains from group B and C PfEMP1, indicating greater antigenic conservation [34–37]; (ii) higher expression levels of group A-like PfEMP1 are associated with severe malaria, lack of antibodies at disease onset, and younger host age [22]; and (iii) antibodies targeting some of these variants were found to be acquired earliest [38–40]. In contrast, uncomplicated malaria involves expression of more polymorphic PfEMP1 variants which, together with clonal antigenic variation, can impede the development of antibodies against the full PfEMP1 repertoire, contributing to recurrent uncomplicated malaria or chronic asymptomatic infections [40]. With increasing age, repeated infections progressively close the gaps in the antibody repertoire and select against parasites expressing previously encountered PfEMP1 variants (reviewed in [41]).

Currently, there is no established gold standard for measuring immunity to malaria or for determining whether a specific antibody effector function confers protection. This is complicated by several factors, including the exposure-dependent acquisition of antibodies, infection-induced fluctuations in antibody levels, the presence of multiple targets with small individual effects, and allelic diversity that requires either a broad antibody repertoire or recognition of conserved epitopes [42]. The latter is particularly important when using PfEMP1 proteins to assess immunity, as these large, multidomain proteins are highly polymorphic, with each parasite strain possessing a unique repertoire of about 60 different variants. We are in the unique position to address this challenge by analyzing samples from a controlled human malaria infection (CHMI) study conducted in Gabon, investigating the infectivity of *P. falciparum* sporozoites (PfSPZ) of the well-characterized reference strain NF54, PfSPZ Challenge (NF54). We hypothesize that differences in naturally acquired, pre-existing, cross-reactive immunity against diverse NF54 PfEMP1 variants underlie the distinct ability of volunteer groups to control CHMI. By measuring strain-specific PfEMP1 antibody levels prior to infection, this study aimed to evaluate their predictive value for CHMI control and to identify the most relevant antigens. Across multiple serological approaches, we found that a broad antibody response, with notable reactivity against B-type PfEMP1, was associated with improved control of infection. This may reflect early recognition of parasites emerging from the liver that express B-type *var* genes [43]. Such an interpretation is consistent with the broader concept of PfEMP1 diversity, whereby parasites expressing novel PfEMP1 variants can evade pre-existing immunity, potentially delaying parasitemia.

## Results

### Grouping participant by low and high susceptibility to the CHMI parasite strain

**Fig 1:**
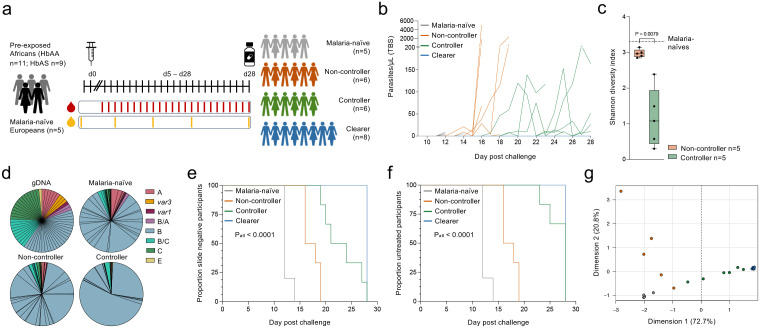
CHMI outcome and clustering of volunteers. **a.** LaCHMI-001 study design and outcome as previously determined [46]. Malaria-naïve Europeans (n = 5) were compared to African individuals with varying history of *falciparum* malaria, which were subgrouped into “non-controller” (n = 6), “controller” (n = 6) and “clearer” (n = 8). The Icons used in the scheme were sourced from https://openclipart.org/. **b.** Growth curves of all individuals included in the LaCHMI-001 trial are shown as parasitemia over time determined by thick blood smear (TBS). **c.** Diversity of *var* gene expression measured by the Shannon diversity index in parasites at the first day of TBS+ in “non-controller” and “controller”. For comparison the mean *var* Shannon index from 9 malaria-naïve volunteers infected with the same number of sporozoites (PfSPZ) [43] is indicated by a horizontal line. A Mann-Whitney U test was used to test for significant difference. **d.** Pie charts showing the genomic distribution of *var* groups and examples of expressed *var* gene patterns in the volunteer groups: malaria-naïve (32.5_C + 11) [43], “non-controller” (L1-008_C + 16) and “controller” (L1-018_C + 28) [46]. **e, f.** Time from challenge infection to parasitemia detection by thick blood smear (TBS) (**e**) and to initiation of treatment (**f**), measured across the different volunteer groups. Rescue treatment before the end of the trial at day 28 was administered either at a parasitemia >1,000 parasites/µL or due to malaria-related symptoms and presence of parasites in the blood. Significant differences between all volunteer groups were assessed using a log-rank test. **g.** Mixed principal component analysis (PCA) and clustering of volunteers based on prepatent period, time to treatment (latency), parasitemia on the day of treatment, and the need for rescue treatment.

During the LaCHMI-001 study, a total of eleven semi-immune Gabonese adults with wild-type hemoglobin (HbAA), nine with sickle cell trait (HbAS), and five malaria-naïve European controls were recruited to undergo CHMI with 3.2x10^3^ PfSPZ of Sanaria PfSPZ Challenge (NF54) via direct venous inoculation (Fig 1a) [44]. All malaria-naïve participants became blood smear positive between days 12–14 (median: day 12) and were immediately treated, as blood smear positivity usually coincides with the onset of symptoms [45] (Fig 1b). In African participants, the prepatent period (defined as the time until thick blood smear (TBS) became parasite-positive) varied between days 14–25 and eight participants remained blood smear negative until the end of the trial on day 28 (termed “clearer”). As previously noted in [46], six volunteers who early turned blood smear positive (median 15.5, range 14–18) developed a parasitemia >1,000 parasites/µL and/or malaria-related symptoms, thereby reaching the predefined treatment criterion (termed “non-controller”). The six remaining volunteers had a longer prepatent period (median 21 days, range 17–25), controlled parasitemia below the treatment threshold of 1,000 parasites/µL, and developed malaria symptoms later or not at all (termed “controller”) (Fig 1b). The classification of volunteers was in line with differences in the diversity of *var* genes expressed in the parasite population recovered from the volunteers (measured as Shannon diversity index) (Fig 1c, 1d). Parasites from non-controllers showed only a slight decrease in *var* expression diversity compared to parasites from malaria-naïve participants [43], whereas parasites from controllers displayed a more uniform, less diverse *var* gene expression pattern [46]. These expression profiles were assessed several days after blood-stage infection, suggesting that the observed differences in *var* gene expression diversity primarily reflect selective pressure from pre-existing blood IgG antibodies, although liver-stage immunity may also restrict the parasite population and thereby influence *var* gene expression diversity. Moreover, to stratify volunteers by susceptibility to the CHMI NF54 strain independently of *var* gene expression, we performed a mixed principal component analysis (PCA) including the prepatent period (Figs 1e, S1a), time to treatment initiation (Figs 1f, S1b), parasitemia at the day of treatment (S1c Fig), and the need for antimalarial treatment. This analysis highlighted the similarity between malaria-naïve and “non-controller” individuals, who together constitute a group with “high susceptibility”. Malaria-naïve individuals were treated immediately after parasites became detectable in the TBS due to their low symptom threshold. The “non-controller” group tolerated higher parasitemia but otherwise behaved similarly. Since parasitemia contributes primarily to the second PCA dimension (S1d Fig), “controllers” and “clearers” – who form a group with “low susceptibility” – would be clearly separated from the “high susceptibility” group along both PCA dimensions (Fig 1g).

### Volunteer groups with high and low susceptibility exhibit different antibody reactivity to full-length PfEMP1 antigens prior to CHMI

**Fig 2:**
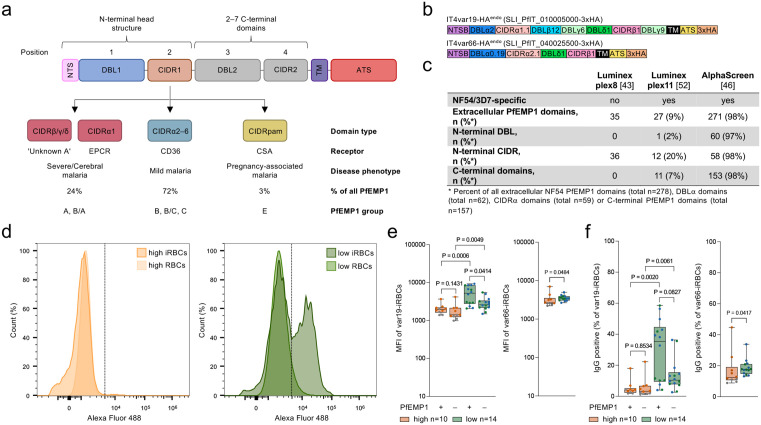
Baseline serorecognition of full-length PfEMP1 anticorrelates with CHMI susceptibility. **a.** Schematic of the PfEMP1 protein domain structure with special emphasis on the N-terminal head structure (NTS-DBLα-CIDR) and their associated binding phenotypes (“Unknown A”, EPCR; CD36, CSA). **b.** Organization of the PfEMP1 domains encoded by the SLI-activated *var* genes IT4var19 (EPCR and ICAM-1 binding) and IT4var66 (CD36 binding) in the cell lines used for the surface serorecognition assay. **c.** Overview of the serologic assays using recombinant PfEMP1 domains applied in the study. The coverage of the NF54-PfEMP1 repertoire is given in percent only for the NF54/3D7-specific assays. **d.** Example of flow cytometry histograms using the IT4var19-HA^endo^ cell line incubated with baseline plasma samples (C-1) from a highsusceptible (L1-015, orange) and lowsusceptible (L1-002, green) individual. Histograms show normalized cell counts (%) over Alexa Fluor 488 intensity for infected (iRBCs) and uninfected red blood cells (RBCs). **e, f.** Quantification of IgG binding from volunteer plasma to IT4var19-HA^endo^-, IT4var19-HA^endo-PfEMP1_neg^ -(PfEMP1 + /–, left panel) and IT4var66-HA^endo^-iRBCs (right panel), shown as mean fluorescence intensity (MFI) (**e**) and as proportion of IgG-positive iRBCs (**f**). Significant differences between volunteer groups were assessed using Mann-Whitney U tests, with multiple comparisons adjusted using the Benjamini-Hochberg procedure. In all panels, combined volunteer groups with high (malaria-naïve n = 5, “non-controller” n = 5) and low susceptibility (“controller” n = 6, “clearer” n = 8) are shown, but individual dots are colored according to the initial volunteer groups with malaria-naïves in grey, “non-controller” in orange, “controller” in green and clearer in blue.

By measuring antibody responses in baseline plasma samples collected prior to CHMI challenge, previously acquired NAI in participating volunteers can be assessed. However, serologic assessment of PfEMP1-specific immunity is not only challenging due to the size and multidomain structure of PfEMP1 proteins (Fig 2a), but also given the protein family’s polymorphic nature resulting in an enormous PfEMP1 repertoire of the parasite population worldwide. To account for this, we applied different experimental strategies targeting either full-length PfEMP1 proteins or individual PfEMP1 domains. Full-length native PfEMP1 were analyzed using a surface recognition FACS assay with parasite lines genetically manipulated to express a single PfEMP1 with defined receptor binding characteristics [47] (Fig 2b). Domain-specific assessments focused either on N-terminal CIDR domains (sequences derived from various parasite strains) associated with particular binding phenotypes (Luminex plex8) or on selected NF54 strain-specific PfEMP1 domains (Luminex plex11) (Fig 2c, S1 Table). In addition, we employed an AlphaScreen assay, which covers nearly the entire PfEMP1 domain repertoire of the NF54 strain and enabling us to explore differences in serorecognition between PfEMP1 groups, domain classes, and the position of domains within full-length proteins (Fig 2c, S1 Table). The AlphaScreen assay is a homogeneous, bead-based assay that enables the detection of protein-protein interactions (i.e., antigen-antibody interactions) without washing steps, making it suitable for high-throughput serological profiling. A key advantage of this platform is that antigens are assayed in a soluble liquid-phase format, which allows antibody binding to occur under conditions that more resemble the native biological environment than solid-phase protein microarrays. In combination with the wheat germ cell-free protein synthesis system (WGCFS), this approach has been increasingly applied to malaria serological studies [48–50]. Of note, no obvious difference in PfEMP1 antibody level was observed between HbAA and HbAS genotypes (S2 Fig); however, a larger sample set may be required to address this question.

First, serorecognition of full-length native PfEMP1 was assessed with three parasite lines. Two lines expressed PfEMP1 variants with distinct N-terminal receptor binding phenotypes: (i) IT4var19-HA^endo^ with dual EPCR and ICAM-1 binding and (ii) IT4var66-HA^endo^ with CD36 binding characteristics (Fig 2a, 2b) [47,51]. As a negative control, a PfEMP1 negative IT4var19-HA^endo-PfEMP1_neg^ was used to validate if the observed reactivity was PfEMP1-dependent. In this cell line PfEMP1 is not detectably expressed on the iRBC surface likely due to reduced surface trafficking [47]. Antibodies contained in plasma samples from volunteers that recognized the surface of the IT4 parasite lines were measured by a flow cytometry-based surface recognition assay (Fig 2d, gating strategy provided in S3 Fig). By plotting the mean fluorescence intensity (MFI) measured for all infected RBCs (Fig 2e) and the proportion of IgG-positive infected RBCs (Fig 2f), differences in surface serorecognition between volunteers with high and low susceptibility were evident for both cell lines. Notably, recognition of iRBCs expressing native IT4VAR19 PfEMP1 more clearly distinguished between the two groups than recognition of parasites expressing the IT4VAR66 PfEMP1 variant. In line with PfEMP1 being the dominant target of the antibody response [9], the PfEMP1 negative IT4var19-HA^endo_PfEMP1_neg^ iRBCs showed strongly reduced serorecognition, albeit differences between the volunteer groups are still visible (Fig 2e, 2f).

### Differences in pre-existing PfEMP1-specific antibody level and breadth between volunteers with high and low susceptibility to CHMI

**Fig 3:**
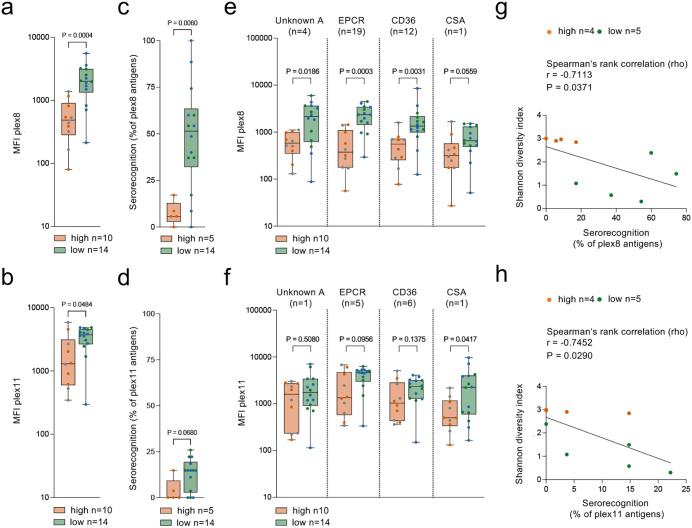
Distinct PfEMP1-specific antibody responses in high- and lowsusceptibility volunteer groups measured by Luminex. **a.–d.** Mean fluorescence intensity (MFI) values averaged across all antigens (**a**, **b**) and percent of serorecognized PfEMP1 antigens (**c**, **d**) were determined for individual volunteer samples and grouped according to susceptibility to NF54 CHMI: high susceptibility (malaria-naïve n = 5, “non-controller” n = 5) and low susceptibility (“controller” n = 6, “clearer” n = 8). Measurements were performed with the baseline plasma samples using Luminex assays targeting 35 N-terminal CIDR domains (plex8) (**a**, **c**) and 27 NF54/3D7-specific PfEMP1 domains (plex11) (**b**, **d**). The mean MFI + 2 standard deviations of malaria–naïve volunteers were used as the cutoff for serorecognition, thus these per definition zero values of malaria–naïve volunteers were excluded from the high susceptibility group for statistical analyses. Significant differences between volunteer groups were assessed using Mann-Whitney U tests. **e, f.** Baseline MFI values for N-terminal CIDR domains recognized by different volunteer groups, stratified by binding phenotypes (“Unknown A”, EPCR, and CD36), are shown for plex8 (**e**) and plex11 (**f**). Each dot represents the average MFI across all antigens within a given antigen group for an individual volunteer sample. Numbers of domains included in each Luminex plex for the respective binding phenotype are indicated above the graph. Differences between volunteer groups were evaluated using the Mann-Whitney U test. **g, h.** Spearman rank correlations were performed between baseline serorecognition measured by Luminex plex8 (**g**) and plex11 (**h**) and the Shannon diversity index of *var* gene expression in parasites from individuals with high (“non-controller”) and low susceptibility (“controller”). In all panels, individual dots are colored according to the initial volunteer groups, with malaria-naïves in grey, “non-controller” in orange, “controller” in green, and “clearer” in blue.

Baseline immunity of all participants was already determined with the Luminex plex8 panel containing 35 N-terminal CIDR domains with defined receptor-binding phenotypes (unknown A, EPCR, CD36, CSA) [46]. Reanalyzing these data using the new clustering of volunteers into high and low susceptibility, significant higher levels of PfEMP1 reactivity measured as mean fluorescence intensity (MFI) and serorecognition (% of recognized antigens) of the different N-terminal CIDR domains (Fig 3a, 3c) were observed for participants of the low susceptibility group. This observation could be confirmed with the NF54-specific Luminex plex11 measuring 27 different PfEMP1 domains. However, plasma from a single malaria-naïve reacted strongly resulting in overall lower serorecognition and discrimination between volunteer groups were lower (Fig 3b, 3d, 3f). The difference between volunteers with high and low susceptibility was evident for all different N-terminal binding phenotypes tested in both Luminex protein panels (Fig 3e, 3f). By plotting the breadth of serorecognition either determined with Luminex plex8 or plex11 against *var* gene expression diversity at the start of infection for people for whom both datasets were available, significant negative correlations were observed (Fig 3g, 3h). This implies that high levels of PfEMP1-specific antibodies shape the parasite population in a way that leads to expression of a reduced *var* gene repertoire.

### Susceptibility to CHMI can be predicted by strain-specific PfEMP1 antibody profile

**Fig 4:**
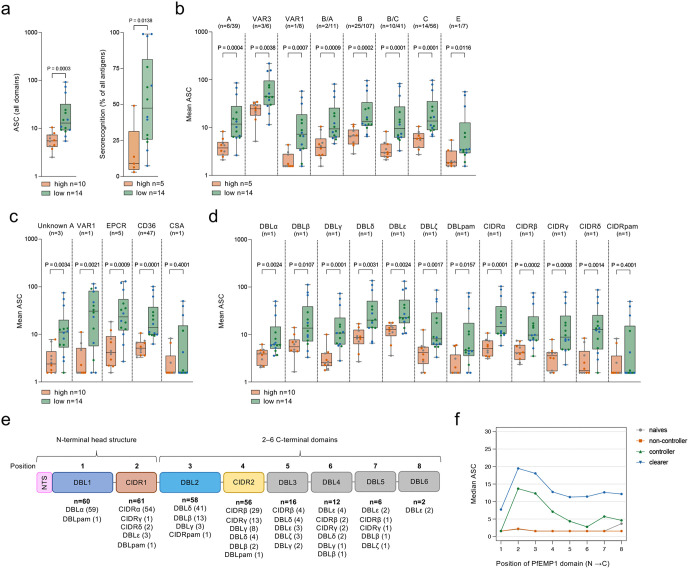
Comprehensive profiling of PfEMP1-specific antibody responses across volunteer groups. **a.** Mean AlphaScreen (ASC) values (left panel) and serorecognition (right panel) for all tested PfEMP1 domains (n = 271) are shown for individual volunteer samples grouped by high and low susceptibility. Differences between groups were assessed using Mann-Whitney U tests. **b–d.** Mean ASC values of PfEMP1 domains measured for individual plasma samples, stratified by PfEMP1 group (**b**), binding phenotype conferred by N-terminal CIDR domains (**c**), major PfEMP1 domain classes (**d**), and volunteer group. Statistical differences between volunteer groups were determined using Mann-Whitney U testing. The number of domains in each category is shown on top of the graph; for *var* groups, the first number refers to the number of genes comprised within the PfEMP1 group, the second number to the number of domains represented on the assay. **e.** PfEMP1 domain architecture and domain composition of the different positions. The total number of domains is shown in bold below each position, while the numbers of domain subtypes are indicated in parentheses. **f.** Median ASC per volunteer group for the different PfEMP1 domain positions 1 to 8. In panels **a–d**, individual dots are colored according to the initial volunteer groups with malaria-naïves in grey, “non-controller” in orange, “controller” in green, and “clearer” in blue.

The African-originating NF54 parasite clone contains 62 different *var* gene copies encoding ten group A, 37 group B, and 14 group C PfEMP1s as well as a single group E variant (VAR2CSA) with a total of 278 different DBL and CIDR domains [19–21]. Given that the Luminex plex11 covers only a small fraction of NF54 PfEMP1 antigens, we expanded our analysis to 98% of all extracellularly exposed NF54 PfEMP1 domains using the AlphaScreen assay, to identify domains possibly relevant for CHMI control (Fig 2c, S1 Table) [52]. Overall, a significant difference in both the mean AlphaScreen Count (ASC) values and the serorecognition across all tested domains was observed between the groups of volunteers with high and low susceptibility (Fig 4a). This pattern remained evident when the data were stratified by PfEMP1 groups (Fig 4b), N-terminal binding phenotypes (Fig 4c), and main domain types (Figs 4d, S4). Interestingly, recognition of the domain at position 1 (DBLα0/1/2 domain present in 98% of PfEMP1 proteins) was markedly lower compared to the subsequent domains at positions 2 (CIDRα domain present in 89% of PfEMP1 proteins) and 3 (DBLδ and DBLβ domains present in 71% and 22% of PfEMP1 proteins, respectively), which showed the highest overall recognition levels (Fig 4e, 4f). This pattern may reflect a genuine biological phenomenon rather than a methodological artifact. However, comparisons of recognition levels across antigens are difficult and influenced by multiple factors including differences in efficiency and quality of recombinant expression, folding, bead coupling and detection of distinct protein domain types.

**Fig 5:**
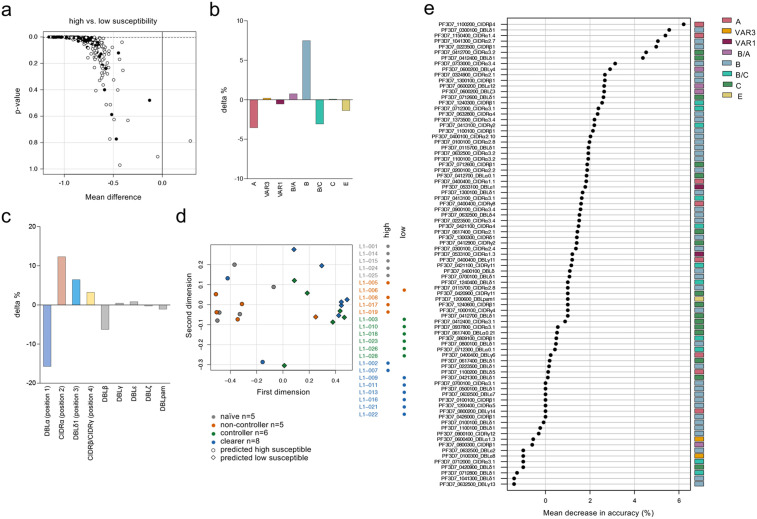
Unsupervised random forest analysis identifying PfEMP1 antigens most strongly associated with high versus low susceptibility in volunteers. **a.** Plasma IgG reactivity of high- versus low-susceptibility volunteer groups to individual PfEMP1 domains (n = 271), measured by AlphaScreen assay, is presented as a volcano plot showing the mean difference versus the p-value obtained from Mann-Whitney U tests. The dashed line indicates a p-value below 0.005. Open circles: DBL domains, black circles: CIDR domains. **b, c.** Difference (in percent) between the observed (% domains found to be most differently recognized) and expected (% domains on assay) proportions of domains identified as most distinct between high- and lowsusceptibility volunteer groups, shown at the *var* group level (**b**) and the domain type level (**c**). A value of 0 indicates that the frequency of a given PfEMP1 group or domain type among the top 83 domains matches its frequency within the PfEMP1 repertoire represented on the assay. **d.** AlphaScreen assay data were analyzed using unsupervised random forest to identify PfEMP1 domain-specific antibodies contributing most to the separation of high- and lowsusceptible volunteer groups. Multidimensional scaling with prediction of the individuals to the pre-defined groups. **e.** The variable importance plot shows the loss of prediction accuracy after random permutation of each variable. The PfEMP1 group of the full-length protein is shown on the right.

To identify PfEMP1 domains whose antibody recognition most strongly contributed to serological separation between volunteers with high and low susceptibility to CHMI, an unsupervised random forest (RF) machine learning approach was applied. AlphaScreen ASC data were z-transformed in order to be able to analyze differences in recognition independent of the level. This showed that all except one domain were recognized at a higher level in volunteers with low susceptibility to CHMI (Fig 5a, S2 Table). Among the 83 domains identified to have the strongest discriminatory signal (p < 0.005, S4c Fig), there was a slight overrepresentation of B-type domains (7.50%) (Fig 5b), as well as CIDRα (position 2), DBLδ1 (position 3), and CIDRβ/γ (position 4) domains (12.33%, 6.48%, and 3.24%, respectively) (Fig 5c), relative to their representation on the assay. Using these 83 most discriminatory domains, the random forest classifier clustered the volunteers into the correct groups that showed 87.5% concordance with susceptibility status, misclassifying only three individuals (Fig 5d). This analysis identified seven domains – three CIDRα, two DBLδ1, and two CIDRβ – from distinct PfEMP1 variants whose seroreactivity contributed most strongly to the separation of the volunteer groups, each causing a > 4% drop in prediction accuracy when randomly permuted (Fig 5e).

### Baseline antibody recognition of PfEMP1 variants associates with lack of expression of the cognate *var* genes

**Fig 6:**
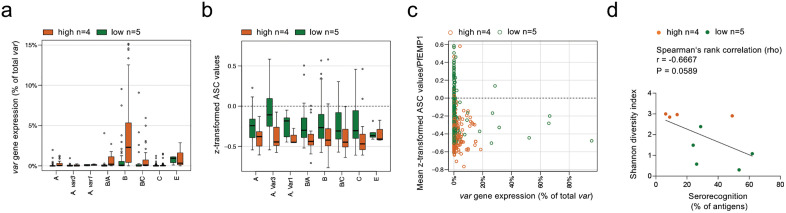
High baseline PfEMP1 antibody levels are associated with absence of *var* gene expression in subsequent CHMI. **a.** Expression of *var* gene groups in “non-controller” (orange, n = 4) and “controller” (green, n = 5) at infection onset. Of note, the y-axis was cut at 16% of total expression, thus single variants from group B or B/C expressed at high level in “controller” are not displayed, but are visible in panel c. **b.** Recognition of the different PfEMP1 groups (A, B/A, B, B/C, C, E/VAR2CSA) and subfamilies (A, VAR3, A, VAR1) displayed as z-transformed ASC values for the subset of “non-controller” (orange, n = 4) and “controller” (green, n = 5) for which RNA data are available. **c.** Plot of PfEMP1 serorecognition (mean of z-transformed ASC values for each domain of individual PfEMP1 variants) versus *var* gene expression (percentage of total *var* expression) shows that only *var* genes encoding PfEMP1s with low baseline serorecognition are expressed by the parasite population. **d.** Non-parametric Spearman rank correlations were performed between baseline serorecognition (% of antigens) of all 271 PfEMP1 antigens measured by AlphaScreen and the Shannon diversity index of *var* gene expression in parasites from individuals with high (“non-controller”) and low susceptibility (“controller”). Serorecognition was calculated by taking the mean of malaria-naïve individuals (n = 5) plus twice the standard deviation (mean + 2xSTD) as a cut-off for serorecognition.

Baseline IgG recognition reflecting previously acquired NAI was broadly elevated across all PfEMP1 domains in volunteers with low susceptibility to CHMI, with B-type domains modestly overrepresented in the serological separation between groups (Fig 5c). This aligns with the predominant expression of B-type *var* genes during early blood-stage infection by NF54 (Fig 6a) [43,44] and suggests that effective control of parasitemia may depend on a broad PfEMP1 antibody repertoire, including the highly polymorphic B-type variants. Such breadth may enable efficient recognition and clearance of iRBCs, thereby limiting both the size of the parasite population (reflected in a longer prepatent period) and the range of *var* genes it expresses. To further explore the relationship between PfEMP1-specific antibody recognition and *var* gene expression, we correlated the *var* gene expression patterns with AlphaScreen recognition in the volunteers for whom RNA data are available, which limited the analysis to nine volunteers (non-controller n = 4, controller n = 5) (Fig 6a, 6b). Plotting the mean z-transformed ASC values for each PfEMP1 variant against the relative expression of the corresponding *var* gene showed that parasites mainly expressed variants with low or absent antibody recognition (negative z-transformed ASC values) (Fig 6c). Stratifying by PfEMP1/*var* group showed that mainly B- and B/C-type of *var* genes were expressed (S5 Fig). When plotting the breadth of serorecognition measured by AlphaScreen against *var* gene expression diversity, a trend toward a negative correlation was observed. (Fig 6d). Together, these findings support a model in which immunity associated with protection from CHMI involve a broad PfEMP1 antibody repertoire spanning the entire PfEMP1 family, with protection potentially being best reflected by the breadth of responses against the highly polymorphic and commonly expressed B-type PfEMP1 variants.

## Discussion

The gradual acquisition of immunity to malaria occurs across a spectrum, encompassing “antidisease immunity” and “antiparasite immunity”, which protects robustly against malaria but almost never results in “sterile immunity” [1]. The first two types of immunity can be linked to two proposed modes by which NAI influences PfEMP1 expression in the infecting parasite population [53]. The first mode describes a reduction in A- and B/A-type *var* gene expression in individuals protected from severe disease, either as the encoded PfEMP1s cause severe malaria and/or are particularly immunogenic. In both cases, these variants elicit immune responses that protect the host during subsequent infections from parasites expressing A- and B/A-type *var* genes, supporting the current model that severe malaria is caused by this relatively conserved subset after a few infections. The second mode refers to the host’s ability to limit the diversity of the non-severity associated PfEMP1 types (B- and C-types) expressed by the infecting parasite population, which is associated with lower parasitemia and better infection control [53]. This more uniform *var* gene expression has been described in a CHMI setting at infection onset for individuals with higher immunity levels [46], as well as in asymptomatic infections [53] and in parasites circulating at the end of the dry season [54]. Thus, this second mode of PfEMP1-affecting immunity can either be acquired during previous infections and cross-react with other parasite strains or can be built up during an ongoing infection, but leads in both cases to a parasite population with less diverse, unified *var* gene expression circulating in the blood. Given that this study focuses on African adults from Lambaréné, Gabon – an area characterized by nearly year-round *P. falciparum* transmission, where more than half of the population has asymptomatic parasitemia, adults infrequently experience symptoms and almost never complications [55,56] – it is reasonable to assume that the African volunteers included in this study possess substantial protection against severe clinical malaria. This is supported by and correlates with the reduction in A-type PfEMP1 expression in comparison to parasites re-isolated from malaria-naïve participants [46]; thus, the first mode of “antidisease immunity” acquisition seems to be already completed in the African volunteers. In line with the current model that immunity to severe malaria is acquired early in life with antibodies to the relatively conserved subset of A- and B/A-type PfEMP1s [41], parasites expressing the DC8-containing B/A-type IT4VAR19 were frequently and at a high level recognized by plasma from semi-immune individuals with low susceptibility to CHMI. Conversely, IgG reactivity to parasites expressing the C-type IT4VAR66 PfEMP1 differed only marginally between the two volunteer groups.

Differences in CHMI control were evident, with a subset of African individuals (“non-controllers”) exhibiting parasite growth nearly resembling that of malaria-naïve participants, albeit symptoms occurred later. These were characterized by rapid blood stage parasite replication, as indicated by short prepatent periods and the need for treatment due to parasitemia exceeding 1,000 parasites/µL or due to symptoms. By grouping malaria-naïve participants and “non-controllers” into one group of “high susceptibility” to CHMI, we were able to compare their antibody responses to those of volunteers who either controlled parasitemia at low levels without developing symptoms (“controller”) or remained blood smear negative (“clearer”) (together grouped into “low susceptibility” to CHMI individuals). This comparison captures differences related to the second mode of PfEMP1-associated immunity acquisition, “antiparasite immunity”, as reflected by the distinct levels of *var* gene expression diversity observed between the two groups. Notably, a similar pattern is observed in natural infections, where symptomatic infections are characterized by a more diverse *var* expression profile than asymptomatic infections. Symptomatic infections are typically recently inoculated, and thus not yet effectively controlled by host immunity [53,54]. Interestingly, volunteer L2-026 showed 60% serorecognition in plex8 but no recognition of NF54-specific antigens in plex11, consistent with a relatively high *var* expression Shannon diversity index of 2.39 and a low prepatent period, albeit being able to control infection at a low parasitemia. This example highlights the importance of strain-specific assays for predicting infection outcomes and suggests that *var* expression diversity could serve as an indicator of strain-specific immunity when measured at the first parasitemia peak in CHMI. Assuming that malaria-naive, non-controller, controller, and clearer individuals represent groups along the continuum of NAI, a larger cohort size would be required to more comprehensively assess differences between these four groups. Such analyses might also reveal clearer hierarchies of PfEMP1 group-specific antibody responses as for example, non-controllers would be expected to recognize disease-associated A- and B/A-type variants at higher levels than malaria-naïve individuals. Nevertheless, the consistency across several independent analyses supports the robustness of the observed associations despite the limited cohort size.

In line with previous studies, our results associate the breadth of antibodies to PfEMP1 variants with infection control [57–60]. A high number of serorecognized antigens was associated with reduced *var* expression diversity, suggesting constraint of the viable *var* gene expression at the onset of infection. CHMI studies in malaria-naïve European volunteers have shown that NF54 parasites are released from the liver with highly similar *var* expression patterns, predominantly expressing a range of B-type *var* genes [43,61]. In semi-immune hosts, parasites expressing PfEMP1 variants recognized by pre-existing antibodies are rapidly eliminated, resulting in a prolonged prepatent period and a reduced *var* expression diversity early in infection [46]. This finding is consistent with the “hole-in-the-wall” theory, which proposes that parasites establish a blood-stage infection by exploiting gaps in the host’s anti-PfEMP1 repertoire [62]. Accordingly, B-type-specific PfEMP1 antibodies appeared proportionally more often among the most discriminatory antibody responses in volunteers with better CHMI control. It is noteworthy that B-type PfEMP1s represent the most polymorphic variants [63,64], which may partly explain why sterile immunity is difficult to achieve. In contrast, *var* genes encoding C- or B/C-type PfEMP1s, located within central chromosomal clusters, are more conserved than other *var* groups. This conservation increases the probability that antibodies elicited during previous infections recognize shared epitopes. Consequently, the presence of antibodies against these variants may primarily reflect cumulative exposure rather than directly mediating improved infection control, particular since these genes are expressed at low frequency in CHMI with the NF54 strain [43,46,61]. Overall, consistent with previous reports, our data support the notion that the gradual acquisition of a broad anti-PfEMP1 antibody repertoire through repeated infections may contribute to “antiparasite immunity” and could facilitate the transition from symptomatic malaria to an asymptomatic carrier state [62]. Similarly, proteins that are either exposed on the merozoite surface or secreted onto its surface during erythrocyte invasion are accessible to antibodies and therefore represent targets of protective immune responses. Although antibody recognition of these antigens was not assessed in the present study, previous work has demonstrated associations with CHMI outcome, e.g., by limiting parasite replication [65–67]. Future studies directly comparing antibody responses to PfEMP1 and merozoite antigens may therefore provide important insights into their relative contributions to naturally acquired immunity.

Direct comparisons of antibody recognition across PfEMP1 domains are inherently challenging due to uncontrolled variations in protein expression, folding, post-translational modification, variable domain occurrence, and differences in sequence diversity. However, by looking at the differences between volunteer groups using z-transformed data, CIDRα1, DBLδ1 and CIDRβ/γ domains at positions 2–4 were overrepresented among the most discriminatory domains, whereas DBLα domains were almost absent. While this enrichment is consistent with the higher frequency of these domains within the PfEMP1 repertoire, it remains unresolved whether it also reflects increased immunogenicity or a greater relevance for protective immunity.

CIDRα1 domains are likely targets of “antidisease immunity” as children with severe malaria lack CIDRα1-specific antibodies during infection but develop them during convalescence which may also explain higher antibody levels observed in uncomplicated malaria cases [38,40,68]. Recently, broadly inhibitory antibodies directed against shared epitopes of EPCR-binding CIDRα1 domains have been isolated from different immune individuals, suggesting that these responses are commonly induced in malaria-exposed individuals [69,70]. Similarly, previous studies have identified DBLβ domains as common targets of IgG antibodies across diverse populations and have been associated with protective immunity [39,71,72]. DBLβ domains can mediate binding to ICAM-1, and PfEMP1 variants exhibiting dual binding to EPCR and ICAM-1 have been implicated in cerebral malaria [14,73,74]. Nevertheless, DBLβ domains did not emerge as significant discriminators between volunteer groups with differing susceptibility to CHMI in our study, nor between malaria-naïve and semi-immune individuals. To definitively establish the protective function of CIDRα1- and/or DBLβ-specific antibodies, longitudinal observational and interventional clinical studies are required to systematically assess expression of PfEMP1 domains and antibody responses across different disease phenotypes. Importantly, such studies should also evaluate antibody effector functions, including C1q binding and FcγR engagement, which may correlate better with protection than total IgG levels alone [72]. Complementary functional assays, such as cytoadhesion/rosetting inhibition or opsonic phagocytosis assays, would further help establish mechanistic links between PfEMP1-specific antibody responses and “antiparasite immunity”, particularly in determining whether these antibodies directly mediate parasite clearance or primarily serve as biomarkers of exposure.

To conclude, our findings indicate that protection associated with “antiparasite immunity” is linked to a broad and diverse antibody repertoire against multiple PfEMP1 domains, including CIDRα across both EPCR- and CD36-binding phenotypes as well as DBLδ1. This breadth suggests that, unlike “antidisease immunity”, “antiparasite immunity” may be inherently more complex and substantially more challenging to achieve through vaccination.

## Materials and methods

### Ethics approval and consent to participate

The study was conducted in accordance with the Declaration of Helsinki (6th revision) and the International Conference on Harmonization-Good Clinical Practice (ICH-GCP) guidelines. The clinical trial in Lambaréné was approved by the Gabonese National Ethics Committee (Comité National d’Éthique de la Recherche) and was filed under a US FDA Investigational New Drug (IND) application. Participant safety was overseen by an independent safety review committee. The trial is registered at ClinicalTrials.gov under identifier NCT02237586 [44]. All volunteers provided written informed consent, and comprehension of the study and procedures was assessed by a quiz. All study participants were adults (18–30 years old). No children or minors were enrolled in this study.

### CHMI trial and blood sampling

Controlled human malaria infections (CHMI) were performed in five malaria-naïve controls and 20 lifelong malaria-exposed African adults in Lambaréné, a region characterized by perennial malaria transmission and semi-immunity to severe disease in adults. Among the 20 African volunteers enrolled in the study, eleven carried the HbAA genotype and nine the HbAS genotype. Prior to CHMI, volunteers were screened for parasitemia by TBS and PCR. To clear potential residual parasites, all participants received a short-acting antimalarial (clindamycin) for 5 days [44], thus all participants were free of asexual parasites one day before CHMI (baseline, C-1). Volunteers were infected by direct venous inoculation (DVI) with 3.2x10^3^ PfSPZ of Sanaria PfSPZ Challenge (NF54). From day 5 post infection, corresponding to the expected onset of merozoite release, daily blood samples were collected for thick smear microscopy and *var* transcription profiling [46]. Plasma samples were obtained, when possible, at baseline on day C-1, and on days C + 7, C + 13, C + 19, and C + 28 post infection. Non-immune volunteers received artemether–lumefantrine as soon as parasites were detected by microscopy. In African adults, treatment was initiated once parasitemia was confirmed by thick smear and malaria-related symptoms were present. Participants with parasitemia >1,000 parasites/µL were treated regardless of symptoms. All volunteers without detectable parasitemia or with persistent low-density, asymptomatic parasitemia were treated on day 28 post infection [44].

### *Var* expression profiling

Analysis of *var* gene expression in parasites re-isolated from volunteers by quantitative real-time PCR was conducted as previously described [43,46].

### Surface recognition assay

Parasite lines IT4var19-HA^endo^, IT4var19-HA^endo-PfEMP1_neg^, and IT4var66-HA^endo^ [47] were grown at 4% hematocrit and 1–2% parasitemia. To obtain 24–30 hpi old parasites, the parasite lines were synchronized with Sorbitol 16 h prior to the assay. Heat-inactivated plasma samples from all volunteers were diluted 1:10 in PBS, added to the red blood cells and incubated for 30 min at room temperature. Cells were washed three times in PBS/2% BSA and stained with 1:200 goat anti-human IgG H + L Alexa488 (10 µg/mL, ThermoFisher), 1:100 DHE (0.005 mg/mL, Biomol/Cayman) and 1:100 Hoechst 33342 (0.005 mg/mL, Biomol/Cayman) for 30 min at room temperature. After 3x washes in PBS/2% BSA, samples were diluted in cold FACS Stop Solution and read in the UV4, B6 and B2 channels of a flow cytometer (Cytek Aurora) until 10,000 iRBCs were measured. A hyperimmune plasma sample was included as a positive control. Non-infected red blood cells (uiRBCs) from parasite cultures stained with plasma samples served as the non-infected control, and plasma from another malaria-naïve donor was used as an additional negative control. Data analysis was performed using FlowJo software. The gating strategy for infected and IgG positive red blood cells is provided in S3 Fig. Data from the malaria-naïve volunteer L1-025 were excluded from this analysis because of consistently high recognition of both uninfected and infected RBCs across all cell lines.

### Luminex assays

Luminex assays using plex8 and plex11 were performed as previously described [46,59]. Plex8 comprises 35 recombinant HIS-tagged N-terminal CIDR domains derived from diverse *P. falciparum* isolates and was designed to broadly capture antibody responses against all main types of PfEMP1s, including those mediating binding to EPCR, CD36, or other yet unidentified host receptors (S1 Table). Plex11 was assembled based on *in vivo var* gene expression data from NF54 parasites in the TüCHMI-001 [43] and LaCHMI-001 [46] studies. The selected proteins cover different NF54 *var* groups and span a range of expression probabilities after liver-stage release (S1 Table). For both plexes, proteins were expressed in Sf9 cells and purified via nickel affinity chromatography. Individual IgG responses were measured in plasma samples from 5 malaria-naïve controls and 19 lifelong malaria-exposed individuals, categorized as non-controllers (n = 5), controllers (n = 6), and clearer (n = 8). Plasma samples were collected at baseline prior to CHMI (day C-1) and diluted 1:80 in Assay Buffer (ABE: 0.1% BSA, 0.05% Tween-20 in PBS, pH 7.4). Assays were conducted by adding 50 µl of bead suspension and 50 µl of diluted plasma to 96-well microtiter plates (MSBVS 1210, Millipore, USA) pre-wetted with ABE. Subsequently, 50 µl of phycoerythrin-conjugated goat anti-human IgG (Jackson ImmunoResearch Laboratories), diluted 1:3500, was added. Mean fluorescence intensities were recorded using the BioPlex100 system. Data from volunteer L1-020 (non-controller) were excluded due to consistently high, non-specific reactivity across nearly all tested antigens (including the negative control BSA) and time points.

### AlphaScreen

AlphaScreen assay was conducted as in [52]. We generated a protein library consisting of 271 PfEMP1 domains derived from 62 *var* genes based on 3D7 strain (S1 Table). Domain boundaries were defined using the VarDom 1.0 server [21], and individual PfEMP1 domains were constructed accordingly. Briefly, the WGCFS translation mixture containing biotinylated recombinant *P. falciparum* proteins (0.1 mL) was diluted 50-fold in reaction buffer (100 mM Tris-HCl, pH 8.0; 0.01% Tween-20; 0.1% bovine serum albumin). Ten microliters of 4,000-fold diluted human sera were added and incubated for 30 min at 26°C to allow formation of immune complexes. Subsequently, streptavidin-coated donor beads (Revvity) and acceptor beads conjugated with protein G (Thermo Scientific) were added to a final concentration of 12 μg/mL each and incubated for 1 h in the dark. The signals were measured using an EnVision plate reader (Revvity). Each assay plate contained a standard curve of biotinylated rabbit IgG, enabling inter-plate standardization using a five-parameter logistic regression model. Standardized values are reported as AlphaScreen counts (ASC). AlphaScreen assays were conducted in a randomized manner to avoid experimental bias.

As also observed in the Luminex assay, plasma from volunteer L1-020 showed consistently high reactivity across nearly all tested antigens and time points, thus was excluded from analysis.

### Statistical data analysis

A Mann-Whitney U test was used to compare the distribution of linear data among two groups. Bonferroni correction was applied if several groups were compared. The Spearman’s rank correlation coefficient was used to assess the relationship between two linear variables. The Shannon index, a measure to assess diversity in communities, was used to estimate diversity of expressed *var* genes in RNA samples. Principal component analysis (PCA) transforms correlated measurements into a set of uncorrelated values to simplify the underlying data structure. For our PCA model, mixed data formats were used, including the prepatent period, the number of days until treatment initiation, parasite counts on the day of treatment and the requirement for rescue treatment, where linear data were z-transformed [*N*(0,1)]. Values of the first two components were plotted to show difference among individuals. An unsupervised random forest (RF) model, a machine learning method based on multiple classification and regression trees, was applied to 83 PfEMP1 domains with AlphaScreen p-values < 0.005 between volunteer groups to estimate proximity among volunteers using the data. Variable importance was calculated, indicating the decrease in prediction accuracy (in percent) when a variable’s values are permuted randomly. Multidimensional scaling was used to visualize clustering of patients. Analyses were performed in GraphPad Prism (version 10.6.1), and *R* (version 4.5.0) using the *randomForest* (version 4.7–1.2) and *PCAmixdata* (version 3.1) packages.

## Supporting information

S1 Fig CHMI outcome. **a.** Time from challenge infection to parasitemia detected by thick blood smear (prepatent period). A horizontal line marks the end of the study on day 28, when all participants received antimalarial treatment. **b.** Day of treatment according to the predefined treatment criteria for malaria-naïve and semi-immune participants. Four “controller” participants received only final treatment on day 28 (horizontal line). **c.** Parasitemia at day of treatment. Of note, malaria-naïve volunteers were treated immediately upon blood smear positivity (thick blood smear), all individuals classified as “non-controllers” required rescue treatment due to malaria-related symptoms or parasitemia above the threshold of 1,000 parasites/µL (indicated by a horizontal line) and “controller” individuals mostly received treatment only at the end of the study (day 28). In panels a–c data from n = 5 malaria-naïve individuals (N, grey), n = 6 “non-controller” (NC, orange) and n = 6 “controller” (C, green) are shown. Significant difference between all volunteer groups was assessed using a Kruskal-Wallis test in each panel. **d.** Squared loading plot showing the contribution of the single variables to the first two dimensions of the PCA shown in Fig 1g.
(TIF)

S2 Fig Impact of hemoglobin genotype on PfEMP1-specific antibody response. **a–c.** Mean fluorescence intensity (MFI) of infected red blood cells expressing IT4var19 and IT4var66 in surface recognition assay (**a**), Luminex plex8 and plex11(**b**), and mean AlphaScreen Counts (ASC) values from AlphaScreen assay (**c**) stained with participants baseline (C-1) plasma samples stratified by hemoglobin genotype. In each panel a non-parametric Mann-Whitney U tests was used to test for statistical differences between both Hb genotypes. In all panels, individual dots are colored according to the initial volunteer groups with “non-controller” in orange, “controller” in green and “clearer” in blue.
(TIF)

S3 Fig FACS gating strategy for surface recognition assay. Gating strategy to discriminate between uninfected (RBCs) and infected red blood cells (iRBCs) based on DHE and Hoechst 33342 staining and to identify IgG positive and negative iRBCs using anti-human IgG labelled with Alexa Fluor 488.
(TIF)

S4 Fig Recognition of subdomain classes of DBLα and CIDRα by volunteer plasma measured by AlphaScreen. **a, b.** AlphaScreen Counts (ASC) for DBLα subdomain classes 0, 1, and 2 (**a**), and CIDRα subdomain classes 1–6 (**b**) for volunteers grouped by high and low susceptibility. The Mann–Whitney U test was used to assess differences between volunteer groups. **c.** Box plots displaying the top 83 significantly differentially recognized PfEMP1 domains with a p-value below 0.005, ranked by recognition difference.
(TIF)

S5 Fig Association of baseline PfEMP1 serorecognition with *var* gene expression at CHMI onset stratified by volunteer groups and PfEMP1/*var* groups. **a, b.** Mean z-transformed ASC across all domains per PfEMP1 variant versus the proportion of *var* gene expression in “controller” (green, n = 5) and “non-controller” (orange, n = 4) are shown for volunteer groups (**a**) and PfEMP1 groups (**b**). For z-transformation of ASC values for each domain (n = 271) data from all volunteers (n = 24) were used, but only the subset of the nine volunteers for which RNA data are available is displayed, leading to a mean of below 0.
(TIF)

S1 Table Overview of serologic assays with PfEMP1 domains included: (a) Luminex plex8, (b) Luminex plex11, (c) AlphaScreen. (XLSX)

S2 Table Data from all serologic assays: (a) Surface serorecognition, (b) Luminex plex8, (c) Luminex plex11, (d) AlphaScreen ASC data, (e) AlphaScreen z-transformed data from top 83 domains. (XLSX)
