# Supplementary figures and images for: Predictive value of PfEMP1 antibody profiles for the course of controlled human malaria infections
Source: PLoS Pathog. 2026 Jun 24;22(6):e1014377. doi: 10.1371/journal.ppat.1014377 (PMC13322502; doi:10.1371/journal.ppat.1014377)

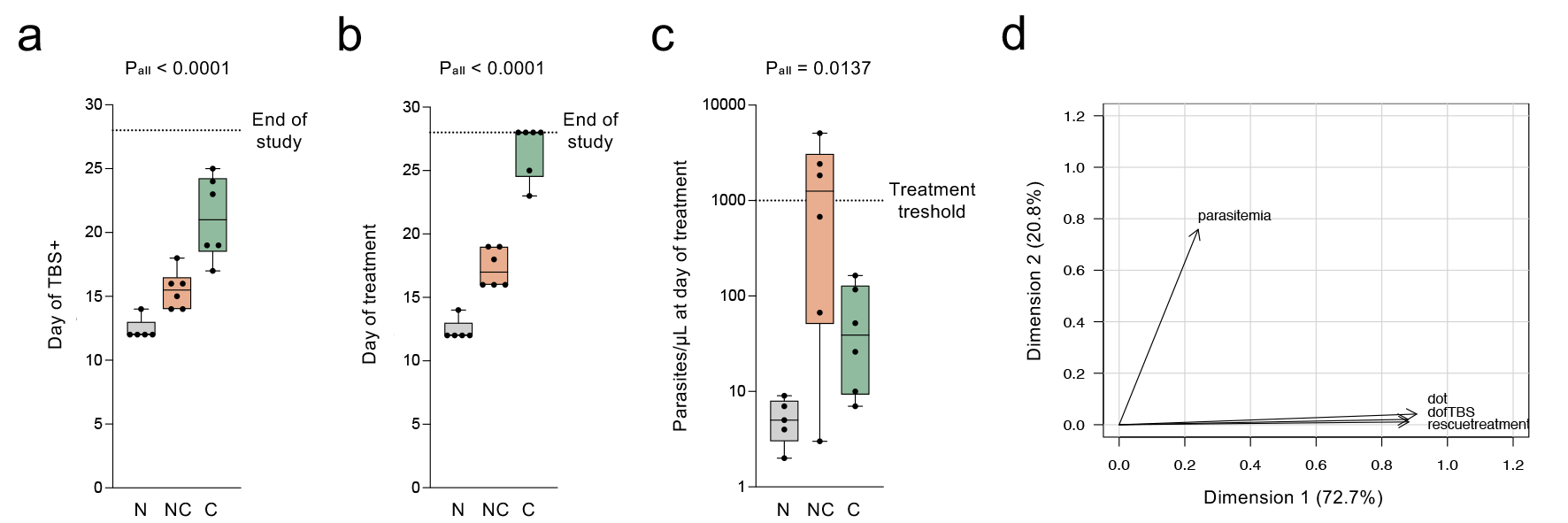

Supplement: S1 Fig — a. Time from challenge infection to parasitemia detected by thick blood smear (prepatent period). A horizontal line marks the end of the study on day 28, when all participants received antimalarial treatment. b. Day of treatment according to the predefined treatment criteria for malaria-naïve and semi-immune participants. Four “controller” participants received only final treatment on day 28 (horizontal line). c. Parasitemia at day of treatment. Of note, malaria-naïve volunteers were treated immediately upon blood smear positivity (thick blood smear), all individuals classified as “non-controllers” required rescue treatment due to malaria-related symptoms or parasitemia above the threshold of 1,000 parasites/µL (indicated by a horizontal line) and “controller” individuals mostly received treatment only at the end of the study (day 28). In panels a–c data from n = 5 malaria-naïve individuals (N, grey), n = 6 “non-controller” (NC, orange) and n = 6 “controller” (C, green) are shown. Significant difference between all volunteer groups was assessed using a Kruskal-Wallis test in each panel. d. Squared loading plot showing the contribution of the single variables to the first two dimensions of the PCA shown in Fig 1g. (TIF) [file ppat.1014377.s001.tif]

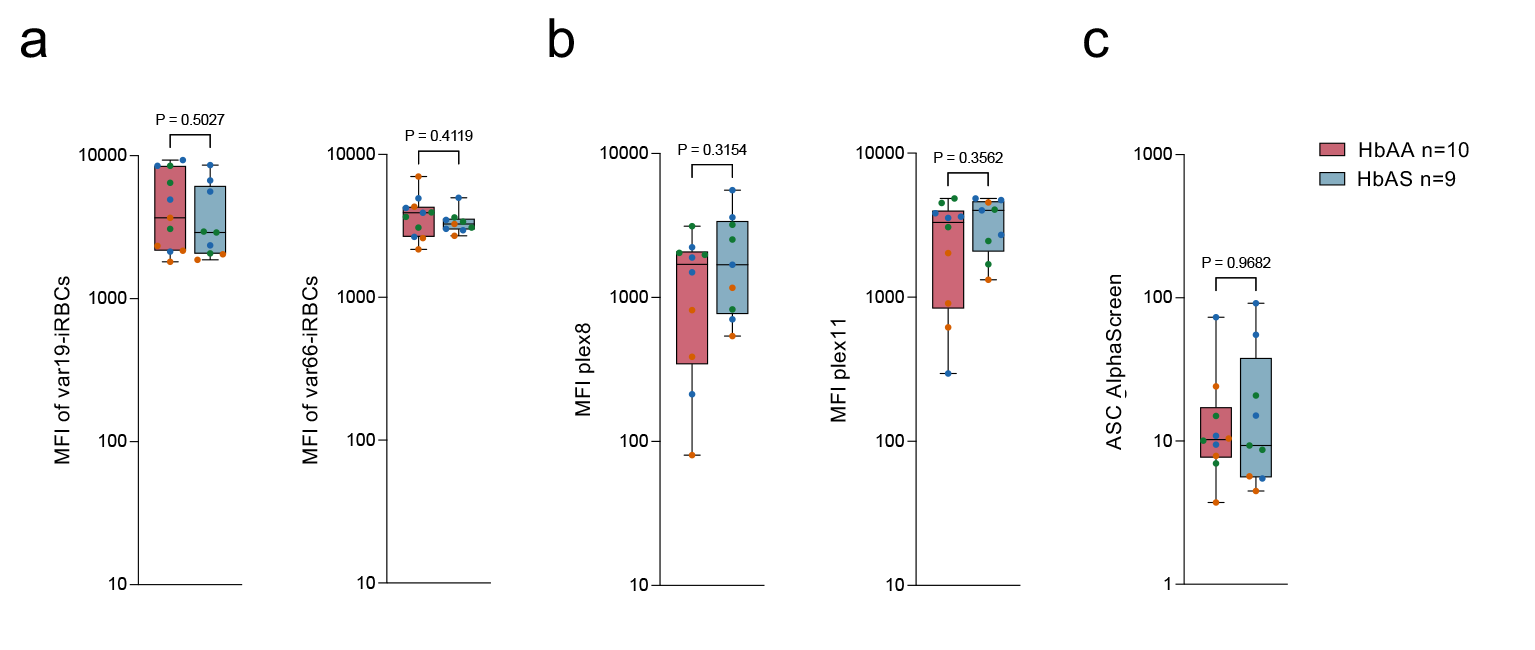

Supplement: S2 Fig — a–c. Mean fluorescence intensity (MFI) of infected red blood cells expressing IT4var19 and IT4var66 in surface recognition assay (a), Luminex plex8 and plex11(b), and mean AlphaScreen Counts (ASC) values from AlphaScreen assay (c) stained with participants baseline (C-1) plasma samples stratified by hemoglobin genotype. In each panel a non-parametric Mann-Whitney U tests was used to test for statistical differences between both Hb genotypes. In all panels, individual dots are colored according to the initial volunteer groups with “non-controller” in orange, “controller” in green and “clearer” in blue. (TIF) [file ppat.1014377.s002.tif]

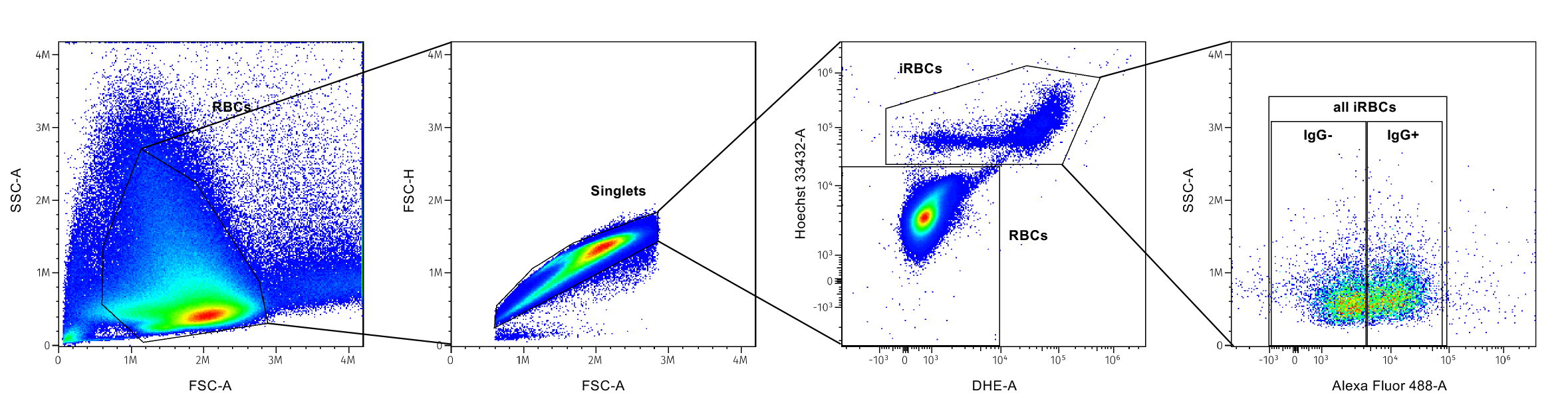

Supplement: S3 Fig — Gating strategy to discriminate between uninfected (RBCs) and infected red blood cells (iRBCs) based on DHE and Hoechst 33342 staining and to identify IgG positive and negative iRBCs using anti-human IgG labelled with Alexa Fluor 488. (TIF) [file ppat.1014377.s003.tif]

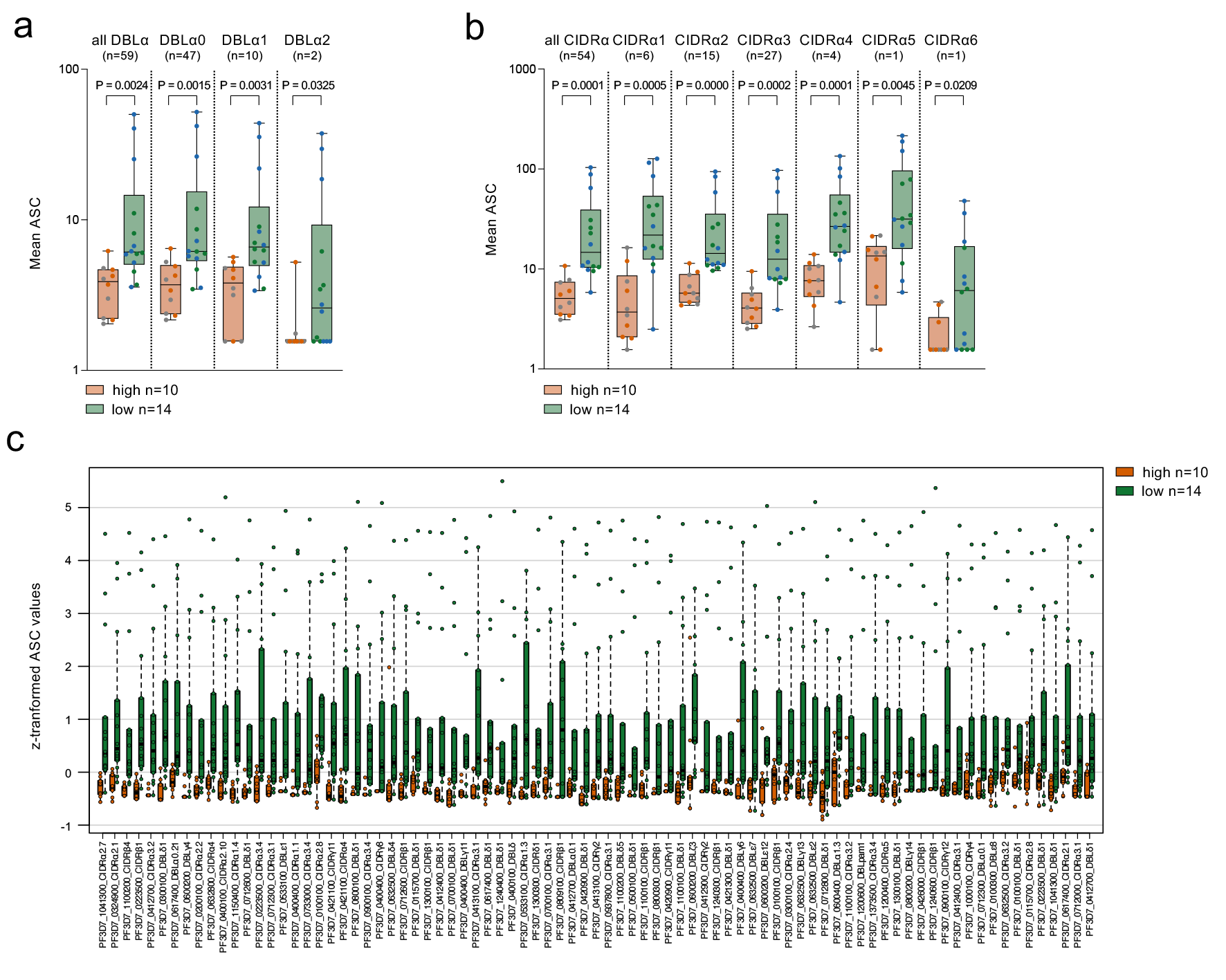

Supplement: S4 Fig — a, b. AlphaScreen Counts (ASC) for DBLα subdomain classes 0, 1, and 2 (a), and CIDRα subdomain classes 1–6 (b) for volunteers grouped by high and low susceptibility. The Mann–Whitney U test was used to assess differences between volunteer groups. c. Box plots displaying the top 83 significantly differentially recognized PfEMP1 domains with a p-value below 0.005, ranked by recognition difference. (TIF) [file ppat.1014377.s004.tif]

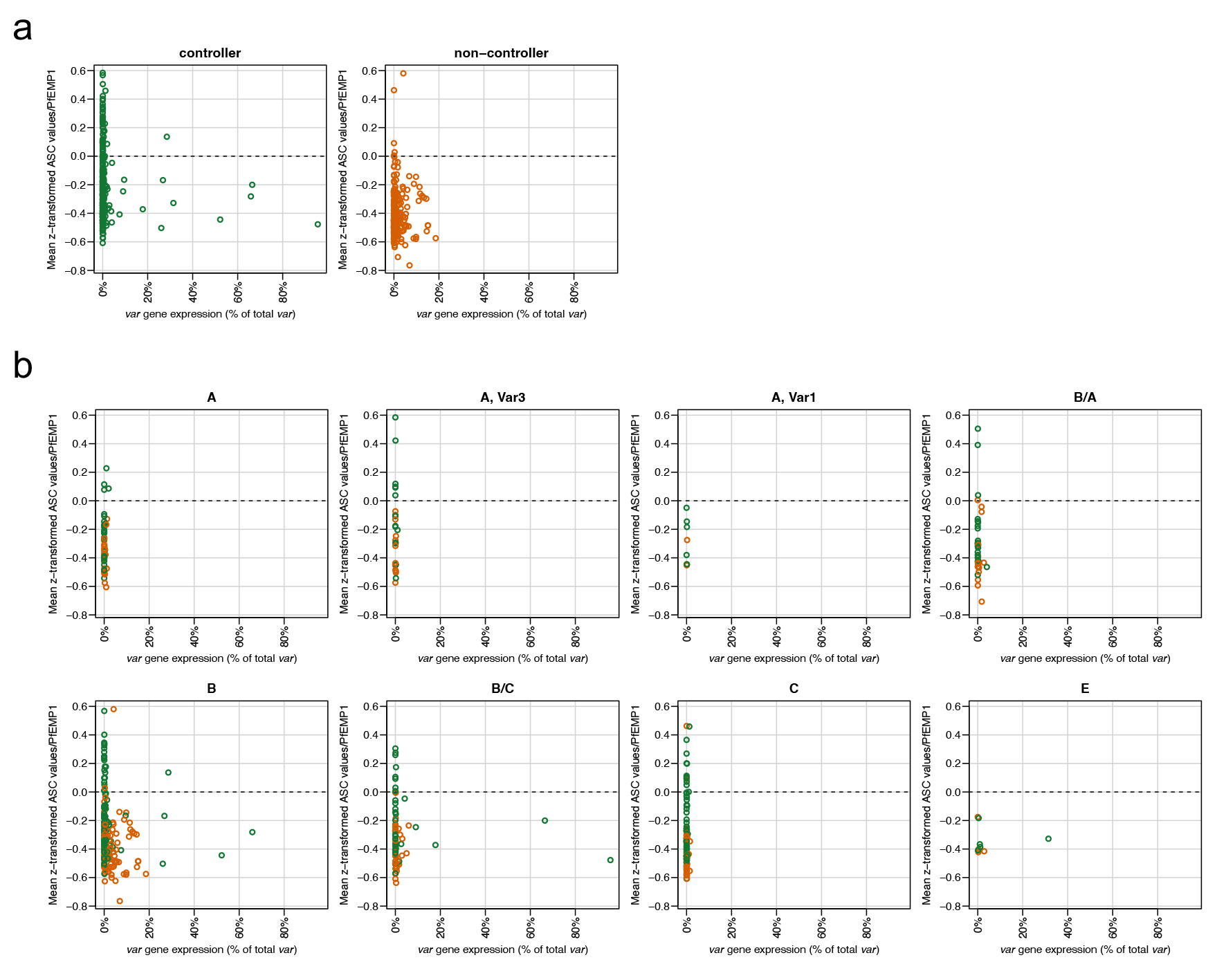

Supplement: S5 Fig — a, b. Mean z-transformed ASC across all domains per PfEMP1 variant versus the proportion of var gene expression in “controller” (green, n = 5) and “non-controller” (orange, n = 4) are shown for volunteer groups (a) and PfEMP1 groups (b). For z-transformation of ASC values for each domain (n = 271) data from all volunteers (n = 24) were used, but only the subset of the nine volunteers for which RNA data are available is displayed, leading to a mean of below 0. (TIF) [file ppat.1014377.s005.tif]
