# Supplementary material for: Predictive value of PfEMP1 antibody profiles for the course of controlled human malaria infections
Source: PLoS Pathog. 2026 Jun 24;22(6):e1014377. doi: 10.1371/journal.ppat.1014377 (PMC13322502; doi:10.1371/journal.ppat.1014377)
Supplement: Attachment — Submitted filename: Response to Reviewers_260522.pdf [file ppat.1014377.s008.pdf]

Response to Reviewers

125 Years BNITM 1900-2025
BERNHARD NOCHT INSTITUTE FOR TROPICAL MEDICINE

Bernhard Nocht Institute for Tropical Medicine
P.O. Box 50 06 40, 22706 Hamburg, Germany

Dr. Anna Bachmann
Junior Group Leader

Bernhard-Nocht-Str. 74
20359 Hamburg, Germany

Phone +49 40 285380-439
Fax +49 40 285380-512
bachmann@bnitm.de

Hamburg, 2026-05-22

Re-submission of paper entitled *Predictive value of PfEMP1 antibody profiles for the course of experimental human malaria infections*

Dear Editors,

We are pleased to submit the revised version of our manuscript entitled *Predictive value of PfEMP1 antibody profiles for the course of experimental human malaria infections* for publication in PLOS Pathogens.

We would like to thank you and the reviewers for the time and effort invested in evaluating our work and for the valuable and constructive feedback provided. In response to the comments received, we have carefully revised the manuscript and addressed all issues raised. A detailed, point-by-point response to the reviewers' comments is provided below.

Best regards,

Dr. Anna Bachmann

Part I – Summary

Please use this section to discuss strengths/weaknesses of study, novelty/significance, general execution and scholarship.

Reviewer #1:

Dr Bachmann and colleagues have performed an in-depth study of antibody responses to PfEMP1 in 20 Gabonese and 5 malaria-naïve adults who participated in a CHMI (controlled human malaria infection) study. The authors have previous published a study (Bachmann et al PLos Pathogens 2019) which uses the same samples to perform a much less detailed serological study, showing that individuals who controlled or cleared infections had higher levels of antibody to a range of PfEMP1 domains (and to some merozoite and other antigens) than those who were malaria naïve and those who failed to control their infections. The present study extends this by (1) including a 3D7 specific multiplex array (2) measuring antibody against almost all 3D7/NF54 PfEMP1 domains by alpha screen and (3) assessing antibody to IEs using two parasite lines with fixed PfEMP1 expression and a control with no surface PfEMP1. This is a very comprehensive assessment of the PfEMP1 antibody response. The overall conclusions from the current study are broadly similar to the first study, but they extend this to show more clearly the importance of antiPfEMP1 antibody as a probable determinant of the types of PfEMP1 expression in parasites that emerge after CHMI challenge.

Reviewer #2:

This study investigated the role of antibodies against PfEMP1, either expressed on the surface of infected erythrocytes or represented by recombinant extracellular domains, in regulating in vivo parasite growth and var gene expression within a controlled human malaria infection (CHMI) model. The cohort comprised five malaria-naïve European adults and twenty malaria-exposed Gabonese adults. All participants were infected with Plasmodium falciparum sporozoites via direct venous inoculation. Based on the prepatent period, participants were stratified into four groups:

- Naïve (European volunteers)
- Non-controllers (prepatent period 14–18 days)
- Controllers (17–25 days)
- Clearers (remained blood-smear negative up to day 28)

For downstream analyses, naïve individuals and non-controllers were grouped as the high-susceptibility group, whereas controllers and clearers were classified as the low-susceptibility group.

Main findings:

- The low-susceptibility group had significantly higher levels of pre-existing, naturally acquired antibodies to:
  1. Native PfEMP1 expressed on the surface of infected erythrocytes
  2. Recombinant proteins representing specific extracellular domains of PfEMP1
- Individuals with antibodies recognizing a broader repertoire of PfEMP1 variants, including group B PfEMP1, demonstrated improved control of parasite growth.
- Parasites from controllers exhibited a more restricted var gene expression profile compared to those from non-controllers and the var diversity was negatively correlated with the breadth of pre-existing naturally acquired antibodies to PfEMP1 variants.

General assessment: The manuscript is well written, and the data are clearly presented. The findings are largely consistent with prior studies, reinforcing existing knowledge. However, the study applies a more exhaustive experimental approach (e.g., AlphaScreen) and novel analytical frameworks, which add value.

Reviewer #3:

This is a novel and conceptually strong manuscript suggesting that pre-existing immunity may influence early parasite antigenic expression, also is highly interesting and well-designed study addressing an important question in malaria immunity. The integration of clinical phenotyping, serology, and parasite gene expression is a major strength. However, the manuscript currently overinterprets correlative data as mechanistic, relies on small sample sizes in critical analyses, lacks validation for predictive models.

Part II – Major Issues: Key Experiments Required for Acceptance

Reviewer #1:

I do not recommend additional experiments

Reviewer #2:

None

Reviewer #3:

The main concern with the conclusions is the overinterpret correlative findings as evidence of protection and mechanism. While the data show an association between broader PfEMP1 specific antibody responses and improved control of parasitemia, there is no direct functional evidence demonstrating that these antibodies mediate parasite clearance, making the use of terms like "protection" and "antiparasite immunity" too strong. In addition, the conclusion emphasizes antibody breadth, but the results also point to the importance of specific domains.

➔ We thank the reviewer for this important and thoughtful comment. We agree that our data are correlative and do not directly demonstrate a causal role of PfEMP1-specific antibodies in parasite clearance. We have therefore revised the manuscript to avoid overinterpretation and to more clearly distinguish association from mechanism.
   - Author summary line 85ff: *Our findings suggest that a broad antibody response contribute to limiting blood-stage infection and highlight the dynamic interplay between parasite antigenic variation and host immunity.*
   - Results line 291ff: *Given that the Luminex plex11 covers only a small fraction of NF54 PfEMP1 antigens, we expanded our analysis to 98% of all extracellularly exposed NF54 PfEMP1 domains using the AlphaScreen assay, to identify domains possibly relevant for CHMI control (Figure 2c) [52].*
   - Results line 330ff: *This aligns with the predominant expression of B-type var genes during early blood-stage infection by NF54 (Figure 6a) [43, 44] and suggests that effective control of parasitemia may depend on a broad PfEMP1 antibody repertoire, including the highly polymorphic B-type variants.*
   - Results line 349ff: *Together, these findings support a model in which immunity associated with protection from CHMI involve a broad PfEMP1 antibody repertoire spanning the entire PfEMP1 family, with protection potentially being best reflected by the breadth of responses against the highly polymorphic and commonly expressed B-type PfEMP1 variants.*
   - Discussion line 435ff: *Overall, consistent with previous reports, our data support the notion that the gradual acquisition of a broad anti-PfEMP1 antibody repertoire through repeated infections may contribute to "antiparasite immunity" and could facilitate the transition from symptomatic malaria to an asymptomatic carrier state [62].*

   In addition, we added a statement highlighting the need for complementary functional assays to establish mechanistic links between PfEMP1-specific antibody responses and parasite clearance.
   - Discussion line 471ff: *Complementary functional assays, such as cytoadhesion/rosetting inhibition or opsonic phagocytosis assays, would further help establish mechanistic links between PfEMP1-specific antibody responses and "antiparasite immunity", particularly in*

determining whether these antibodies directly mediate parasite clearance or primarily serve as biomarkers of exposure.

However, we are convinced that the importance of overall antibody breadth and magnitude is well supported by the data, whereas conclusions regarding the specific contribution of individual domains should be interpreted more cautiously. First, only a slight overrepresentation of B-type domains (7.5%) was observed among the most discriminatory antigens, a point also raised by another reviewer. Second, although domains located at positions 2 and 3 (particularly CIDRα, DBLδ, and CIDRβ domains) appeared to be recognized at higher levels, direct comparisons between different antigens remain challenging for several reasons discussed in line 301ff and line 445ff. Third, the seven antigens identified as contributing most strongly to the discrimination between volunteer groups resulted in only modest reductions in prediction performance following random permutation (4.4–6.2%). For these reasons, we highlighted potentially interesting findings at the single-domain level while carefully contextualizing their interpretation throughout the manuscript.

Part III – Minor Issues: Editorial and Data Presentation Modifications

Please use this section for editorial suggestions as well as relatively minor modifications of existing data that would enhance clarity.

<u>Reviewer #1:</u>

Major comments:

1. Whilst it is clear that the presence of higher levels of PfEMP1 antibody correlate with lower breadth of PfEMP1 expression (and one can infer a causative link here), it is not as clear that the PfEMP1 antibodies are what drives the outcomes of challenge in the study participants. Certainly increasing breadth and strength of PfEMP1 response appears to correlate with outcomes, but isn't it equally possible that the relationship between PfEMP1 antibody and outcome could be mediated through greater immunity to merozoite antigens, limiting parasite replication? The authors imply causation, and they are possible correct (e.g. lines 281-3), but they need to be open to other explanations.

➔ We are grateful for this comment and are pleased to include additional discussion on this aspect, which we have also considered extensively in the context of other studies assessing both recognition of merozoites (e.g., using the mADRB assay) and recognition of PfEMP1 (Wichers et al., 2021). In brief, we believe that there is a strong correlation between the presence of antibodies directed against merozoite antigens and PfEMP1 recognition; however, this hypothesis requires further thorough investigation in larger sample cohorts. Nevertheless, we have added paragraphs to the Introduction and Discussion sections, also incorporating the recently accepted paper from Faith Osier's group on the identification of merozoite antigens associated with clinical immunity in CHMI.

- Introduction line 104ff: *The main targets of the blood stage antibody response are merozoite surface and secreted antigens as well as the variant surface antigen P. falciparum erythrocyte membrane protein 1 (PfEMP1), the key virulence factor mediating immune evasion [6-9].*
- Discussion line 438ff: *Similarly, proteins that are either exposed on the merozoite surface or secreted onto its surface during erythrocyte invasion are accessible to antibodies and therefore represent targets of protective immune responses. Although antibody recognition of these antigens was not assessed in the present study, previous work has demonstrated associations with CHMI outcome, e.g., by limiting parasite replication [65-67]. Future studies directly comparing antibody responses to PfEMP1 and merozoite antigens may therefore provide important insights into their relative contributions to naturally acquired immunity.*

2. In Figure 4 the authors refer to their profiling as "systems serology". This is a misuse of the term, which refers to functional and/or biophysical profiling of antibody responses, of the sort the authors refer to at the end of the discussion.

➔ We agree and have corrected the title of Figure 4: *Comprehensive profiling of PfEMP1-specific antibody responses across volunteer groups.*

3. In terms of relative importance of each PfEMP1 group to immunity, it seems from the Random Forest that of the seven most important features, their break down by group (A, B, C) is not unexpected or favouring any one group- two A three B and two C; B are about half the 3D7 PfEMP1s. Most of the 83 differently detected proteins (Figure 5e) are making pretty tiny contributions to the accuracy of classification.

➔ To our understanding, even the seven domains associated with the highest loss in prediction accuracy individually accounted for only 4.4–6.2% of the model performance reduction (Table S2), which is comparatively modest relative to other studies screening for relevant antigens (e.g., Ogwang et al., 2026). These findings suggest that broad antibody recognition, rather than responses to a small number of individual antigens, is associated with improved control of parasitemia.
Nevertheless, among the 83 most significantly recognized domains, there was a slight overrepresentation of B-type PfEMP1 domains (+7.5%). Importantly, the vast majority of domains were differentially recognized (191 out of 271), but only those 83 with a p-value < 0.005 were included in the random forest analysis and analyzed in Figure 5B and C. Accordingly, we cautiously stated to observe a "*slight overrepresentation*" of B-types and concluded that our "*these findings support a model in which immunity associated with protection from CHMI involve a broad PfEMP1 antibody repertoire spanning the entire PfEMP1 family, with protection potentially being best reflected by the breadth of responses against the highly polymorphic and commonly expressed B-type PfEMP1 variants.*" (Results, line 316ff, line 349ff). This interpretation further considers that the var gene expression profile observed in NF54 parasites at the onset of blood-stage infection is clearly dominated by B-type var genes. Consequently, individuals capable of recognizing a broader range of B-type PfEMP1 domains may be better able to control infection early during parasite expansion. At the same time, telomeric B-type var genes are the most polymorphic members of the var gene family. Therefore, prior exposure to these specific NF54-derived B-type PfEMP1 domains would actually be less likely than exposure to the more conserved A- or C-type PfEMP1 variants. In this context, it was somewhat unexpected that any distinct pattern emerged at all.

4. In the text (lines 267-269) the authors state that representation differs from what would be expected by chance. It is not clear- is this "0" if all domains are equally represented? Some aid to interpretation is needed for the reader.

➔ Yes, a value of 0 indicates that the proportion of domains from a given var/PfEMP1 group among the top 83 domains corresponds exactly to its proportion within the genome represented on the assay. We added an explanation to the Figure 5 legend and slightly revised the corresponding sentence in the Results section to improve clarity:
   – Figure 5 legend: b, c. *Difference (in percent) between the observed (% domains found to be most differently recognized) and expected (% domains on assay) proportions of domains identified as most distinct between high- and low-susceptibility volunteer groups, shown at the var group level (c) and the domain level (d). A value of 0 indicates that the frequency of a given PfEMP1 group or domain type among the top 83 domains matches its frequency within the PfEMP1 repertoire represented on the assay.*
   – Results line 316ff: *Among the 83 domains identified to have the strongest discriminatory signal (p < 0.005, Figure S4c), there was a slight overrepresentation of B-type domains (7.50%) (Figure 5b), as well as CIDRα (position 2), DBLδ1 (position 3), and CIDRβ/γ (position 4) domains (12.33%, 6.48%, and 3.24%, respectively) (Figure 5c), relative to their representation on the assay.*

5. Given the relatively small sample size (24 individuals, after censoring) the robustness of the analysis needs to be raised as a potential shortcoming.

➔ We acknowledge that the relatively small sample size represents a limitation of the study and may reduce statistical power, particularly for subgroup analyses (naïve, non-controller, controller, clearer). At the same time, the controlled CHMI setting and the consistency of several independent analyses support the robustness of the observed associations despite the limited cohort size. We appreciate the reviewer raising this important point, which we have now explicitly addressed it in the discussion.

- Discussion line 407ff: *Assuming that malaria-naive, non-controller, controller, and clearer individuals represent groups along the continuum of NAI, a larger cohort size would be required to more comprehensively assess differences between these four groups. Such analyses might also reveal clearer hierarchies of PfEMP1 group-specific antibody responses as for example, non-controllers would be expected to recognize disease-associated A- and B/A-type variants at higher levels than malaria-naïve individuals. Nevertheless, the consistency across several independent analyses supports the robustness of the observed associations despite the limited cohort size.*

Minor comments:

1. My understanding and my experience is that the IT4VAR19 parasite line binds EPCR but NOT ICAM-1, it has a CIDRalpha 1.1 domain and is a DC8 type variant.

➔ It has recently been shown that IT4VAR19-expressing parasites are capable of binding ICAM-1, which is correctly referenced at the end of the sentence in line 256 with Crohnshagen et al., 2025 (reference 47). We have now additionally included McLean et al., 2025 (reference 51), which provides further evidence that DC8 might confer dual binding to EPCR and ICAM-1. We agree that this observation was unexpected and intensively discussed with the authors of Crohnshagen et al. 2025. However, it is also supported by AlphaFold predictions, which are largely consistent with a potential ICAM-1 binding capacity of the DBLβ12 domain. Further details can be found in the online version of the dissertation from Jakob Crohnshagen: https://ediss.sub.uni-hamburg.de/handle/ediss/10924.

2. Line 255-257: I do not understand this terminology e.g. 98% DBLalpha 0/1/2, how is this lower than the 89% CIDRalpha?

➔ Thanks for pointing this out. These numbers refer to the domain type present on this position, not to recognition. To improve clarity, we have revised and expanded the descriptions accordingly.

- Line 298ff: *Interestingly, recognition of the domain at position 1 (DBLα0/1/2 domain present in 98% of PfEMP1 proteins) was markedly lower compared to the subsequent domains at positions 2 (CIDRα domain present in 89% of PfEMP1 proteins) and 3 (DBLδ and DBLβ domains present in 71% and 22% of PfEMP1 proteins, respectively), which showed the highest overall recognition levels (Figure 4e, f).*
- Figure legend 4: *e. PfEMP1 domain architecture and domain composition of the different positions. The total number of domains is shown in bold below each position, while the numbers of domain subtypes are indicated in parentheses.*

3. In Figure 3 e and f and Figure 4 it is not clear what the numbers in brackets ref to e.g. (n=1) or (n=14/56)

➔ In both figures, these numbers refer to the domains included in the assay. In Figure 4b, the first number indicates the number of var genes within the respective var group, and the second number indicates the number of domains represented on the assay (e.g., n = 14/56 refers to 14 group C var genes represented by 56 domains on the assay). We have added or revised the corresponding descriptions in the respective figure legends for clarity.

- Figure 3 e, f legend: *Numbers of domains included in each Luminex plex for the respective binding phenotype are indicated above the graph.*

- Figure 4: *The number of domains in each category is shown on top of the graph; for var groups, the first number refers to the number of genes comprised within the PfEMP1 group, the second number to the number of domains represented on the assay.*

<u>Reviewer #2:</u>

Overall comments:

1. Since var expression is dependent on antibody levels, I suggest antibody levels be on the x-axis and var expression on the y-axis.

➔ We agree that axis assignment can reflect an implicit directional interpretation, and that antibody levels are more appropriately considered as the independent variable in this context. We have therefore updated the figures 3 (g & h) and 6 (panels c & d) accordingly, placing antibody levels on the x-axis and var expression on the y-axis.

2. The concept of naturally acquired immunity should be more explicitly emphasized, as the baseline antibody measurements reflect immune exposure acquired through natural infection and are central to interpreting CHMI outcomes

➔ We agree and have introduced this concept when baseline plasma samples are first mentioned. See also later comment on the result section.
   - Results line 230f: *By measuring antibody responses in baseline plasma samples collected prior to CHMI challenge, previously acquired NAI in participating volunteers can be assessed.*

   Additionally, we clarified this the time point of plasma sample collection in the abstract:
   - Abstract line 42ff: *In this study, different serological assays targeting both full-length native PfEMP1 and individual domains was applied to plasma samples obtained prior to challenge infection at study baseline from 25 individuals enrolled in a CHMI study of PfSPZ Challenge (NF54), that included malaria-naïve Europeans and lifelong malaria-exposed Africans with varying degrees of immunity. All assays showed strong predictive value of both PfEMP1 antibody levels and breadth at study baseline for CHMI outcome.*

3. The AlphaScreen assay is introduced as though it is widely familiar. A brief definition and explanation would improve accessibility for readers.

➔ We explained the assay now briefly when first mentioned in the results section starting.
   - Results line 243ff: *The AlphaScreen assay is a homogeneous, bead-based assay that enables the detection of protein-protein interactions (i.e., antigen-antibody interactions) without washing steps, making it suitable for high-throughput serological profiling. A key advantage of this platform is that antigens are assayed in a soluble liquid-phase format, which allows antibody binding to occur under conditions that more resemble the native biological environment than solid-phase protein microarrays. In combination with the wheat germ cell-free protein synthesis system (WGCFS), this approach has been increasingly applied to malaria serological studies [48-50].*

Specific comments:

1. Abstract

- The term “AlphaScreen data” is not intuitive and may be difficult for readers to interpret. Consider rephrasing including paraphrasing to capture the assay

➔ We thank the reviewer for this helpful suggestion. We agree that the term “AlphaScreen data” may not be sufficiently descriptive for all readers. We have therefore revised the wording to more clearly describe the assay and the type of measurements performed (recognition of PfEMP1 domains by antibodies present in plasma at study baseline measured by AlphaScreen).
   - Abstract line 47ff: *A random forest machine learning analysis of antibody recognition profiles, measured by AlphaScreen nearly covering all extracellular PfEMP1 domains of the NF54-CHMI*

parasite strain, suggested that a broad antibody repertoire, including antibodies against the most diversified B-type PfEMP1 domains, represents a discriminative feature distinguishing volunteers with high versus low susceptibility to CHMI.

– Lines 46–48: "All assays showed strong predictive value…" This statement is vague. It would be more informative to explicitly state whether recognition of homologous or heterologous PfEMP1 domains showed stronger predictive performance.

➔ We thank the reviewer for this comment. As the random forest prediction analysis was based exclusively on homologous PfEMP1 domain recognition, we feel that directly comparing predictive performance between homologous and heterologous antigens would go beyond the scope of the current analysis. However, we agree that this distinction is important and have thus discussed an apparent difference of homologous versus heterologous antigen recognition in a single patient. We therefore decided not to include this point in the abstract.

2. Introduction

– Lines 92–94: The concept of mutually exclusive var gene expression has been challenged by recent work (PMID: 40379932). This should be incorporated to reflect current understanding.

➔ We agree and modified the section accordingly.

- Introduction line 110ff: *Each parasite genome is equipped with about 60 var genes encoding different PfEMP1 variants. While var gene expression has long been considered mutually exclusive, with a single dominant PfEMP1 variant expressed on the surface of the infected red blood cell (iRBC), recent work suggests that var expression may be more flexible than previously thought, and that co-expression of multiple var genes can occur at the single-cell level [12].*

– Lines 133–136: "In contrast uncomplicated malaria...". The is not unclear. uncomplicated malaria is associated with less diverse var expression, but this statement seems to suggest otherwise. Clarify the intended distinction.

➔ We greatly appreciate this comment, as it highlights an important potential source of confusion between diversity in var gene expression and sequence-level polymorphism. We have therefore revised the sentence to clarify the intended meaning more precisely.

- Introduction line 157ff: *In contrast, uncomplicated malaria involves expression of more polymorphic PfEMP1 variants which, together with clonal antigenic variation, can impede the development of antibodies against the full PfEMP1 repertoire, contributing to recurrent uncomplicated malaria or chronic asymptomatic infections [40].*

3. Results

– Line 189: Define TBS (thick blood smear) at first mention.

➔ Done.

– Line 276: Clarify that baseline antibody levels represent "naturally acquired immunity"

➔ We agree and have revised the first sentence of the paragraph to address this comment. See also previous general comment.

- Results line 328ff: *Baseline IgG recognition reflecting previously acquired NAI was broadly elevated across all PfEMP1 domains in volunteers with low susceptibility to CHMI, with B-type domains modestly overrepresented in the serological separation between groups (Figure 5c).*

4. Figures

– Figure 1e–f: Specify the statistical test used to calculate p-values. Given the time-to-event nature of the data, Cox regression would be appropriate, but it is not clear whether this was applied.

➔ Significant differences between all volunteer groups were assessed using a log-rank test, as stated in the figure legend. The Log-Rank Test is a standard test for determining whether survival

distributions differ significantly. Since we are comparing only two groups and not accounting for additional confounding variables, we refrained from fitting a full Cox regression model.

– Figure 2: Panels d and e are difficult to interpret. In the key, replace “low” and “high” with “low-susceptibility” and “high-susceptibility” for clarity. Or make it more explicit in the figure legend as done in Figure 3 legend.

➔ We have added a more detailed description to the figure legend for clarity.
   – Figure 2 legend: *In all panels, combined volunteer groups with high (malaria-naïve n=5, "non-controller" n=5) and low susceptibility ("controller" n=6, "clearer" n=8) are shown, but individual dots are colored according to the initial volunteer groups with malaria-naïves in grey, "non-controller" in orange, "controller" in green and clearer in blue.*

Reviewer #3:

1. The manuscript is generally well presented, but several minor issues could improve clarity. The definition of serorecognition based on a naïve mean + 2 SD cutoff is somewhat arbitrary and may influence breadth estimates; a sensitivity analysis or presentation of continuous data would strengthen confidence in the results.

➔ We agree that 2SD cutoff is arbitrary and could influence breadth estimates. To assess this, we have conducted a sensitivity analysis, by comparing of the +2 SD cutoff (left plot, as shown in the manuscript) with a more lenient and stringent cutoff of mean naïve controls (1.5 and + 3 SDs + 4 STD, right plots) for the Luminex plex8 data set is shown below. The group differences remain statistically significantly different. For this reason, we have kept the mean naïve + 2 SD cutoff in order to remain consistent with previous publications. Also, as suggested continuous MFI/ASC values are already shown alongside the serorecognition plots in Figure 3 (plex8: panel a, plex11: panel b) and 4a, albeit averaged across all antigens or antigens within the antigen group. This has now been clarified in figure legends.

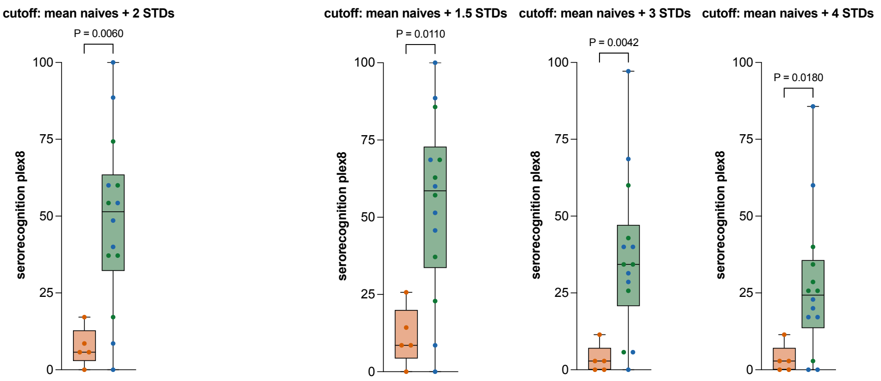

   – Figure 3 legend: ***a.–d.*** *Mean fluorescence intensity (MFI) values averaged across all antigens (**a, b**) and percent of serorecognized PfEMP1 antigens (**c, d**) were determined for individual volunteer samples and grouped according to susceptibility to NF54 CHMI: high susceptibility (malaria-naïve n=5, "non-controller" n=5) and low susceptibility ("controller" n=6, "clearer" n=8). Measurements were performed with the baseline plasma samples using Luminex assays targeting 35 N-terminal CIDR domains (plex8) (**a, c**) and 27 NF54/3D7-specific PfEMP1 domains (plex11). (**b, d**). The mean MFI + 2 standard deviations of malaria–naïve volunteers were used as the cutoff for serorecognition, thus these per definition zero values of malaria–naïve volunteers were excluded from the high susceptibility group for statistical analyses. Significant differences between volunteer groups were assessed using Mann-Whitney U tests.* ***e, f.*** *Baseline MFI values for N-terminal CIDR domains recognized by different volunteer groups, stratified by binding phenotypes ("Unknown A", EPCR, and CD36), are shown for plex8 (**e**) and plex11 (**f**). Each*

dot represents the average MFI across all antigens within a given antigen group for an individual volunteer sample. Numbers of domains included in each Luminex plex for the respective binding phenotype are indicated above the graph. Differences between volunteer groups were evaluated using the Mann-Whitney U test. ***g, h.*** *Spearman rank correlations were performed between baseline serorecognition measured by Luminex plex8 (**g**) and plex11 (**h**) and the Shannon diversity index of var gene expression in parasites from individuals with high ("non-controller") and low susceptibility ("controller"). In all panels, individual dots are colored according to the initial volunteer groups, with malaria-naïves in grey, "non-controller" in orange, "controller" in green, and "clearer" in blue.*

2. Introduction: Line 155-157. It is more appropriate to suggest that this pattern may reflect early recognition of parasites expressing B-type var genes upon emergence from the liver. This interpretation aligns with the broader concept of PfEMP1 diversity, where parasites expressing novel variants can evade pre-existing immunity, potentially delaying parasitemia.

➔ We agree and changed the two sentences accordingly.

- Introduction line 179ff: *This may reflect early recognition of parasites emerging from the liver that express B-type var genes [43]. Such an interpretation is consistent with the broader concept of PfEMP1 diversity, whereby parasites expressing novel PfEMP1 variants can evade pre-existing immunity, potentially delaying parasitemia.*
