# Supplementary material for: Predictive value of PfEMP1 antibody profiles for the course of controlled human malaria infections
Source: PLoS Pathog. 2026 Jun 24;22(6):e1014377. doi: 10.1371/journal.ppat.1014377 (PMC13322502; doi:10.1371/journal.ppat.1014377)
Supplement: Peer review history

# Peer review: Decision Letter 0

**DOI:** 10.1371/journal.ppat.1014377.r001

**Authors:** Marcelo U. Ferreira, Tracey J. Lamb

6 May 2026

PPATHOGENS-D-26-00578

Predictive value of PfEMP1 antibody profiles for the course of controlled human malaria infections

PLOS Pathogens

Dear Dr. Bachmann,

Thank you for submitting your manuscript to PLOS Pathogens. After careful consideration, we feel that it has merit but does not fully meet PLOS Pathogens's publication criteria as it currently stands. Therefore, we invite you to submit a revised version of the manuscript that addresses the points raised during the review process.

Please submit your revised manuscript by Jul 05 2026 11:59PM. If you will need more time than this to complete your revisions, please reply to this message or contact the journal office at plospathogens@plos.org. When you're ready to submit your revision, log on to https://www.editorialmanager.com/ppathogens/ and select the 'Submissions Needing Revision' folder to locate your manuscript file.

Please include the following items when submitting your revised manuscript:

* A letter that responds to each point raised by the editor and reviewer(s). You should upload this letter as a separate file labeled 'Response to Reviewers'. This file does not need to include responses to any formatting updates and technical items listed in the 'Journal Requirements' section below.

* A marked-up copy of your manuscript that highlights changes made to the original version. You should upload this as a separate file labeled 'Revised Manuscript with Track Changes'.

* An unmarked version of your revised paper without tracked changes. You should upload this as a separate file labeled 'Manuscript'.

If you would like to make changes to your financial disclosure, competing interests statement, or data availability statement, please make these updates within the submission form at the time of resubmission. Guidelines for resubmitting your figure files are available below the reviewer comments at the end of this letter.

As the corresponding author, your ORCID iD is verified in the submission system and will appear in the published article. PLOS supports the use of ORCID, and we encourage all coauthors to register for an ORCID iD and use it as well. Please encourage your coauthors to verify their ORCID iD within the submission system before final acceptance, as unverified ORCID iDs will not appear in the published article. Only the individual author can complete the verification step; PLOS staff cannot verify ORCID iDs on behalf of authors.

We look forward to receiving your revised manuscript.

Kind regards,

Marcelo U. Ferreira, MD, PhD

Academic Editor

PLOS Pathogens

Tracey Lamb

Section Editor

PLOS Pathogens

Sumita Bhaduri-McIntosh

Editor-in-Chief

PLOS Pathogens

orcid.org/0000-0003-2946-9497

Michael Malim

Editor-in-Chief

PLOS Pathogens

orcid.org/0000-0002-7699-2064

**Journal Requirements:**

1) Please ensure that the CRediT author contributions listed for every co-author are completed accurately and in full.

At this stage, the following Authors/Authors require contributions: Yannick D. Höppner, Ralf Krumkamp, Hikaru Nagaoka, Charlotte Wapler, Hannah Honner, Louise Turner, Jean Claude Dejon-Agobé, Yabo J. Honkpehedji, Jeannot Fréjus Zinsou, Jana Held, Meral Esen, Heidrun von Thien, Iris Bruchhaus, B. Kim Lee Sim, Stephen L. Hoffman, Peter G. Kremsner, Tim-Wolf Gilberger, Bertrand Lell, Takafumi Tsuboi, Thomas Lavstsen, Eizo Takashima, Benjamin Mordmüller, and Anna Bachmann. Please ensure that the full contributions of each author are acknowledged in the "Add/Edit/Remove Authors" section of our submission form.

The list of CRediT author contributions may be found here: https://journals.plos.org/plospathogens/s/authorship#loc-author-contributions

2) We ask that a manuscript source file is provided at Revision. Please upload your manuscript file as a .doc, .docx, .rtf or .tex. If you are providing a .tex file, please upload it under the item type u2018LaTeX Source Fileu2019 and leave your .pdf version as the item type u2018Manuscriptu2019.

3) We do not publish any copyright or trademark symbols that usually accompany proprietary names, eg ©,  ®, or TM  (e.g. next to drug or reagent names). Therefore please remove all instances of trademark/copyright symbols throughout the text, including:

- ® on pages: 6, and 16.

4) Please upload all main figures as separate Figure files in .tif or .eps format. For more information about how to convert and format your figure files please see our guidelines:

https://journals.plos.org/plospathogens/s/figures

5) We notice that your supplementary Figures are included in the manuscript file. Please remove them and upload them with the file type 'Supporting Information'. Please ensure that each Supporting Information file has a legend listed in the manuscript after the references list.

6) Some material included in your submission may be copyrighted. According to PLOS’s copyright policy, authors who use figures or other material (e.g., graphics, clipart, maps) from another author or copyright holder must demonstrate or obtain permission to publish this material under the Creative Commons Attribution 4.0 International (CC BY 4.0) License used by PLOS journals. Please closely review the details of PLOS’s copyright requirements here: PLOS Licenses and Copyright. If you need to request permissions from a copyright holder, you may use PLOS's Copyright Content Permission form.

Please respond directly to this email and provide any known details concerning your material's license terms and permissions required for reuse, even if you have not yet obtained copyright permissions or are unsure of your material's copyright compatibility. Once you have responded and addressed all other outstanding technical requirements, you may resubmit your manuscript within Editorial Manager.

Potential Copyright Issues:

Figure 1a. Please confirm whether you drew the images / clip-art within the figure panels by hand. If you did not draw the images, please provide (a) a link to the source of the images or icons and their license / terms of use; or (b) written permission from the copyright holder to publish the images or icons under our CC BY 4.0 license. Alternatively, you may replace the images with open source alternatives. See these open source resources you may use to replace images / clip-art:

- https://commons.wikimedia.org

- https://openclipart.org/

Note: If the reviewer comments include a recommendation to cite specific previously published works, please review and evaluate these publications to determine whether they are relevant and should be cited. There is no requirement to cite these works unless the editor has indicated otherwise.

**Reviewers' Comments:**

Reviewer's Responses to Questions

**Part I - Summary**

Please use this section to discuss strengths/weaknesses of study, novelty/significance, general execution and scholarship.

Reviewer #1: Dr Bachmann and colleagues have performed an in-depth study of antibody responses to PfEMP1 in 20 Gabonese and 5 malaria-naïve adults who participated in a CHMI (controlled human malaria infection) study. The authors have previous published a study (Bachmann et al PLos Pathogens 2019) which uses the same samples to perform a much less detailed serological study, showing that individuals who controlled or cleared infections had higher levels of antibody to a range of PfEMP1 domains (and to some merozoite and other antigens) than those who were malaria naïve and those who failed to control their infections. The present study extends this by (1) including a 3D7 specific multiplex array (2) measuring antibody against almost all 3D7/NF54 PfEMP1 domains by alpha screen and (3) assessing antibody to IEs using two parasite lines with fixed PfEMP1 expression and a control with no surface PfEMP1. This is a very comprehensive assessment of the PfEMP1 antibody response.

The overall conclusions from the current study are broadly similar to the first study, but they extend this to show more clearly the importance of antiPfEMP1 antibody as a probable determinant of the types of PfEMP1 expression in parasites that emerge after CHMI challenge.

Reviewer #2: Study summary

This study investigated the role of antibodies against PfEMP1, either expressed on the surface of infected erythrocytes or represented by recombinant extracellular domains, in regulating in vivo parasite growth and var gene expression within a controlled human malaria infection (CHMI) model. The cohort comprised five malaria-naïve European adults and twenty malaria-exposed Gabonese adults.

All participants were infected with Plasmodium falciparum sporozoites via direct venous inoculation. Based on the prepatent period, participants were stratified into four groups:

• Naïve (European volunteers)

• Non-controllers (prepatent period 14–18 days)

• Controllers (17–25 days)

• Clearers (remained blood-smear negative up to day 28)

For downstream analyses, naïve individuals and non-controllers were grouped as the high-susceptibility group, whereas controllers and clearers were classified as the low-susceptibility group.

Main findings

• The low-susceptibility group had significantly higher levels of pre-existing, naturally acquired antibodies to:

1. Native PfEMP1 expressed on the surface of infected erythrocytes

2. Recombinant proteins representing specific extracellular domains of PfEMP1

• Individuals with antibodies recognizing a broader repertoire of PfEMP1 variants, including group B PfEMP1, demonstrated improved control of parasite growth.

• Parasites from controllers exhibited a more restricted var gene expression profile compared to those from non-controllers and the var diversity was negatively correlated with the breadth of pre-existing naturally acquired antibodies to PfEMP1 variants.

• General assessment

The manuscript is well written, and the data are clearly presented. The findings are largely consistent with prior studies, reinforcing existing knowledge. However, the study applies a more exhaustive experimental approach (e.g. AlphaScreen) and novel analytical frameworks, which add value.

Overall comments

• The concept of naturally acquired immunity should be more explicitly emphasized, as the baseline antibody measurements reflect immune exposure acquired through natural infection and are central to interpreting CHMI outcomes

The AlphaScreen assay is introduced as though it is widely familiar. A brief definition and explanation would improve accessibility for readers.

Specific comments:

Abstract

• The term “AlphaScreen data” is not intuitive and may be difficult for readers to interpret. Consider rephrasing including paraphrasing to capture the assay

• Lines 46–48: “All assays showed strong predictive value…”

This statement is vague. It would be more informative to explicitly state whether recognition of homologous or heterologous PfEMP1 domains showed stronger predictive performance.

Introduction

• Lines 92–94: The concept of mutually exclusive var gene expression has been challenged by recent work (PMID: 40379932). This should be incorporated to reflect current understanding.

• Lines 133–136: “In contrast uncomplicated malaria...”. The is not unclear. uncomplicated malaria is associated with less diverse var expression, but this statement seems to suggest otherwise. Clarify the intended distinction.

Results

• Line 189: Define TBS (thick blood smear) at first mention.

• Line 276: Clarify that baseline antibody levels represent “naturally acquired immunity”.

Figures

• Figure 1e–f: Specify the statistical test used to calculate p-values. Given the time-to-event nature of the data, Cox regression would be appropriate, but it is not clear whether this was applied.

• Figure 2: Panels d and e are difficult to interpret. In the key, replace “low” and “high” with “low-susceptibility” and “high-susceptibility” for clarity. Or make it more explicit in the figure legend as done in Figure 3 legend.

Reviewer #3: This is a novel and conceptually strong manuscript suggesting that pre-existing immunity may influence early parasite antigenic expression, also is highly interesting and well-designed study addressing an important question in malaria immunity. The integration of clinical phenotyping, serology, and parasite gene expression is a major strength. However, the manuscript currently: overinterprets correlative data as mechanistic, relies on small sample sizes in critical analyses, lacks validation for predictive models

**********

**Part II – Major Issues: Key Experiments Required for Acceptance**

Please use this section to detail the key new experiments or modifications of existing experiments that should be absolutely required to validate study conclusions.

Generally, there should be no more than 3 such required experiments or major modifications for a "Major Revision" recommendation. If more than 3 experiments are necessary to validate the study conclusions, then you are encouraged to recommend "Reject".

Reviewer #1: I do not recommend additional experiments

Reviewer #2: none

Reviewer #3: The main concern with the conclusions is the overinterpret correlative findings as evidence of protection and mechanism. While the data show an association between broader PfEMP1 specific antibody responses and improved control of parasitemia, there is no direct functional evidence demonstrating that these antibodies mediate parasite clearance, making the use of terms like “protection” and “antiparasite immunity” too strong. In addition, the conclusion emphasizes antibody breadth, but the results also point to the importance of specific domains.

**********

**Part III – Minor Issues: Editorial and Data Presentation Modifications**

Please use this section for editorial suggestions as well as relatively minor modifications of existing data that would enhance clarity.

Reviewer #1: MAJOR COMMENTS:

1. Whilst it is clear that the presence of higher levels of PfEMP1 antibody correlate with lower breadth of PfEMP1 expression (and one can infer a causative link here), it is not as clear that the PfEMP1 antibodies are what drives the outcomes of challenge in the study participants. Certainly increasing breadth and strength of PfEMP1 response appears to correlate with outcomes, but isn’t it equally possible that the relationship between PfEMP1 antibody and outcome could be mediated through greater immunity to merozoite antigens, limiting parasite replication? The authors imply causation, and they are possible correct (e.g. lines 281-3), but they need to be open to other explanations.

2. In Figure 4 the authors refer to their profiling as “systems serology”. This is a misuse of the term, which refers to functional and/or biophysical profiling of antibody responses, of the sort the authors refer to at the end of the discussion.

3. In terms of relative importance of each PfEMP1 group to immunity, it seems from the Random Forest that of the seven most important features, their break down by group (A, B,C) is not unexpected or favouring any one group- two A three B and two C; B are about half the 3D7 PfEMP1s. Most of the 83 differently detected proteins (Figure 5e) are making pretty tiny contributions to the accuracy of classification.

4. In the text (lines 267-269) the authors state that representation differs from what would be expected by chance. It is not clear- is this “0” if all domains are equally represented? Some aid to interpretation is needed for the reader.

5. Given the relatively small sample size (24 individuals, after censoring) the robustness of the analysis needs to be raised as a potential shortcoming.

Minor comments

1. My understanding and my experience is that the IT4VAR19 parasite line binds EPCR but NOT ICAM-1, it has a CIDRalpha 1.1 domain and is a DC8 type variant.

2. Line 255-257: I do not understand this terminology e.g. 98% DBLalpha 0/1/2, how is this lower than the 89% CIDRalpha?

3. In Figure 3 e and f and Figure 4 it is not clear what the numbers in brackets ref to e.g. (n=1) or (n=14/56)

Reviewer #2: Since var expression is dependent on antibody levels, I suggest antibody levels be on the x-axis and var expression on the y-axis.

Reviewer #3: The manuscript is generally well presented, but several minor issues could improve clarity. The definition of serorecognition based on a naïve mean + 2 SD cutoff is somewhat arbitrary and may influence breadth estimates; a sensitivity analysis or presentation of continuous data would strengthen confidence in the results.

Introduction: Line 155-157. It is more appropriate to suggest that this pattern may reflect early recognition of parasites expressing B-type var genes upon emergence from the liver. This interpretation aligns with the broader concept of PfEMP1 diversity, where parasites expressing novel variants can evade pre-existing immunity, potentially delaying parasitemia.

**********

PLOS authors have the option to publish the peer review history of their article (what does this mean?). If published, this will include your full peer review and any attached files.

If you choose “no”, your identity will remain anonymous but your review may still be made public.

**Do you want your identity to be public for this peer review?**  For information about this choice, including consent withdrawal, please see our Privacy Policy.

Reviewer #1: No

Reviewer #2: No

Reviewer #3: No

[NOTE: If reviewer comments were submitted as an attachment file, they will be attached to this email and accessible via the submission site. Please log into your account, locate the manuscript record, and check for the action link "View Attachments". If this link does not appear, there are no attachment files.]

**Figure resubmission:**

-->While revising your submission, we strongly recommend that you use PLOS’s NAAS tool (https://ngplosjournals.pagemajik.ai/artanalysis) to test your figure files. NAAS can convert your figure files to the TIFF file type and meet basic requirements (such as print size, resolution), or provide you with a report on issues that do not meet our requirements and that NAAS cannot fix.

After uploading your figures to PLOS’s NAAS tool - https://ngplosjournals.pagemajik.ai/artanalysis, NAAS will process the files provided and display the results in the "Uploaded Files" section of the page as the processing is complete. If the uploaded figures meet our requirements (or NAAS is able to fix the files to meet our requirements), the figure will be marked as "fixed" above. If NAAS is unable to fix the files, a red "failed" label will appear above. When NAAS has confirmed that the figure files meet our requirements, please download the file via the download option, and include these NAAS processed figure files when submitting your revised manuscript.

**Reproducibility:**

To enhance the reproducibility of your results, we recommend that authors of applicable studies deposit laboratory protocols in protocols.io, where a protocol can be assigned its own identifier (DOI) such that it can be cited independently in the future. Additionally, PLOS ONE offers an option to publish peer-reviewed clinical study protocols. Read more information on sharing protocols at https://plos.org/protocols?utm_medium=editorial-email&utm_source=authorletters&utm_campaign=protocols

# Author comment: Author response to Decision Letter 1

**DOI:** 10.1371/journal.ppat.1014377.r002

25 May 2026

Attachment Submitted filename: Response to Reviewers_260522.pdf

# Peer review: Decision Letter 1

**DOI:** 10.1371/journal.ppat.1014377.r003

**Authors:** Marcelo U. Ferreira, Tracey J. Lamb

12 Jun 2026

Dear Dr. Bachmann,

We are pleased to inform you that your manuscript 'Predictive value of PfEMP1 antibody profiles for the course of controlled human malaria infections' has been provisionally accepted for publication in PLOS Pathogens.

Before your manuscript can be formally accepted you will need to complete some formatting changes, which you will receive in a follow up email. A member of our team will be in touch with a set of requests.

Please note that your manuscript will not be scheduled for publication until you have made the required changes, so a swift response is appreciated.

IMPORTANT: The editorial review process is now complete. PLOS will only permit corrections to spelling, formatting or significant scientific errors from this point onwards. Requests for major changes, or any which affect the scientific understanding of your work, will cause delays to the publication date of your manuscript.

Should you, your institution's press office or the journal office choose to press release your paper, you will automatically be opted out of early publication. We ask that you notify us now if you or your institution is planning to press release the article. All press must be co-ordinated with PLOS.

Thank you again for supporting Open Access publishing; we are looking forward to publishing your work in PLOS Pathogens.

Best regards,

Marcelo U. Ferreira, MD, PhD

Academic Editor

PLOS Pathogens

Tracey Lamb

Section Editor

PLOS Pathogens

Sumita Bhaduri-McIntosh

Editor-in-Chief

PLOS Pathogens

orcid.org/0000-0003-2946-9497

Michael Malim

Editor-in-Chief

PLOS Pathogens

orcid.org/0000-0002-7699-2064

***********************************************************

Reviewer Comments (if any, and for reference):

Reviewer's Responses to Questions

**Part I - Summary**

Please use this section to discuss strengths/weaknesses of study, novelty/significance, general execution and scholarship.

Reviewer #1: The authors have revised their manuscript and taken all my suggestions into account.

Reviewer #2: as provided in my initial review report

Reviewer #3: This is a well-conducted and informative study that provides novel insights into PfEMP1-specific antibody responses and parasite control during controlled human malaria infection. The manuscript is well written, the analyses are appropriate, and the conclusions are generally supported by the data.

**********

**Part II – Major Issues: Key Experiments Required for Acceptance**

Please use this section to detail the key new experiments or modifications of existing experiments that should be absolutely required to validate study conclusions.

Generally, there should be no more than 3 such required experiments or major modifications for a "Major Revision" recommendation. If more than 3 experiments are necessary to validate the study conclusions, then you are encouraged to recommend "Reject".

Reviewer #1: No experiments required

Reviewer #2: all previous concerns have been adequately addressed

Reviewer #3: Thank you for addressing my previous concern regarding the interpretation of the antibody findings. The revised text now more appropriately frames the results as associations rather than direct evidence of protective mechanisms. I also appreciate the inclusion of specific PfEMP1 domains alongside the discussion of antibody breadth, which provides a more balanced interpretation of the findings. I have no further major concerns regarding this point.

**********

**Part III – Minor Issues: Editorial and Data Presentation Modifications**

Please use this section for editorial suggestions as well as relatively minor modifications of existing data that would enhance clarity.

Reviewer #1: no further issues

Reviewer #2: none

Reviewer #3: I would mark my previous comments as resolved.

**********

PLOS authors have the option to publish the peer review history of their article (what does this mean?). If published, this will include your full peer review and any attached files.

If you choose “no”, your identity will remain anonymous but your review may still be made public.

**Do you want your identity to be public for this peer review?**  For information about this choice, including consent withdrawal, please see our Privacy Policy.

Reviewer #1: No

Reviewer #2: No

Reviewer #3: No

# Editor report: Acceptance letter

**DOI:** 10.1371/journal.ppat.1014377.r004

**Authors:** Marcelo U. Ferreira, Tracey J. Lamb

Dear Dr. Bachmann,

We are delighted to inform you that your manuscript, "

Predictive value of PfEMP1 antibody profiles for the course of controlled human malaria infections," has been formally accepted for publication in PLOS Pathogens.

We have now passed your article onto the PLOS Production Department who will complete the rest of the pre-publication process. All authors will receive a confirmation email upon publication.

The corresponding author will soon be receiving a typeset proof for review, to ensure errors have not been introduced during production. Please review the PDF proof of your manuscript carefully, as this is the last chance to correct any scientific or type-setting errors. Please note that major changes, or those which affect the scientific understanding of the work, will likely cause delays to the publication date of your manuscript. Note: Proofs for Front Matter articles (Pearls, Reviews, Opinions, etc...) are generated on a different schedule and may not be made available as quickly.

Soon after your final files are uploaded, the early version of your manuscript, if you opted to have an early version of your article, will be published online. The date of the early version will be your article's publication date. The final article will be published to the same URL, and all versions of the paper will be accessible to readers.

For Research Articles, you will receive an invoice from PLOS for your publication fee after your manuscript has reached the completed accept phase. If you receive an email requesting payment before acceptance or for any other service, this may be a phishing scheme. Learn how to identify phishing emails and protect your accounts at https://explore.plos.org/phishing.

Thank you again for supporting open-access publishing; we are looking forward to publishing your work in PLOS Pathogens.

Best regards,

Sumita Bhaduri-McIntosh

Editor-in-Chief

PLOS Pathogens

orcid.org/0000-0003-2946-9497

Michael Malim

Editor-in-Chief

PLOS Pathogens

orcid.org/0000-0002-7699-2064
